# Preserving full spectrum information in imaging mass spectrometry data reduction

**DOI:** 10.1093/bioinformatics/btaf247

**Published:** 2025-05-08

**Authors:** Roger A R Moens, Lukasz G Migas, Jacqueline M Van Ardenne, Eric P Skaar, Jeffrey M Spraggins, Raf Van de Plas

**Affiliations:** Delft Center for Systems and Control, Delft University of Technology, 2628 CD Delft, Zuid-Holland, The Netherlands; Delft Center for Systems and Control, Delft University of Technology, 2628 CD Delft, Zuid-Holland, The Netherlands; Mass Spectrometry Research Center, Vanderbilt University, Nashville, TN 37232, United States; Department of Chemistry, Vanderbilt University, Nashville, TN 37232, United States; Department of Pathology, Microbiology, and Immunology, Vanderbilt University Medical Center, Nashville, TN 37232, United States; Mass Spectrometry Research Center, Vanderbilt University, Nashville, TN 37232, United States; Department of Chemistry, Vanderbilt University, Nashville, TN 37232, United States; Department of Pathology, Microbiology, and Immunology, Vanderbilt University Medical Center, Nashville, TN 37232, United States; Department of Cell and Developmental Biology, Vanderbilt University, Nashville, TN 37232, United States; Department of Biochemistry, Vanderbilt University, Nashville, TN 37232, United States; Delft Center for Systems and Control, Delft University of Technology, 2628 CD Delft, Zuid-Holland, The Netherlands; Mass Spectrometry Research Center, Vanderbilt University, Nashville, TN 37232, United States; Department of Biochemistry, Vanderbilt University, Nashville, TN 37232, United States

## Abstract

**Motivation:**

Imaging mass spectrometry (IMS) has become an important tool for molecular characterization of biological tissue. However, IMS experiments tend to yield large datasets, routinely recording over 200 000 ion intensity values per mass spectrum and more than 100 000 pixels, i.e. spectra, per dataset. Traditionally, IMS data size challenges have been addressed by feature selection or extraction, such as by peak picking and peak integration. Selective data reduction techniques such as peak picking only retain certain parts of a mass spectrum, and often these describe only medium-to-high-abundance species. Since lower-intensity peaks and, for example, near-isobar species are sometimes missed, selective methods can potentially bias downstream analysis toward a subset of species in the data rather than considering all species measured.

**Results:**

We present an alternative to selective data reduction of IMS data that achieves similar data size reduction while better conserving the ion intensity profiles across all recorded *m/z*-bins, thereby preserving full spectrum information. Our method utilizes a low-rank matrix completion model combined with a randomized sparse-format-aware algorithm to approximate IMS datasets. This representation offers reduced dimensionality and a data footprint comparable to peak picking but also captures complete spectral profiles, enabling comprehensive analysis and compression. We demonstrate improved preservation of lower signal-to-noise ratio signals and near-isobars, mitigation of selection bias, and reduced information loss compared to current state-of-the-art data reduction methods in IMS.

**Availability and implementation:**

The source code is available at https://github.com/vandeplaslab/full_profile and data are available at https://doi.org/10.4121/a6efd47a-b4ec-493e-a742-70e8a369f788.

## 1 Introduction

Imaging mass spectrometry (IMS) is an analytical imaging technology that enables molecular mapping of complex biological samples, such as tissues, biofilms, or dispersed cells (Caprioli *et al.* 1997, McDonnell and Heeren 2007, Bien *et al.* 2022, Perry *et al.* 2022, Esselman *et al.* 2023). IMS combines the sensitivity and specificity of mass spectrometry with spatial information. It enables researchers to concurrently measure the distribution of hundreds to thousands of molecular species throughout tissue sections or other heterogeneous samples without the need for labeling target molecules (Caprioli *et al.* 1997, McDonnell and Heeren 2007, Aichler and Walch 2015, Buchberger *et al.* 2018). This capability holds strong potential for probing the lipidomic, glycomic, metabolomic, and proteomic content of biological samples across a wide range of applications, spanning from fundamental research in biology and medicine to the development of novel diagnostics and therapeutics (Rubakhin *et al.* 2005, Kaspar *et al.* 2011, Vaysse *et al.* 2017).

However, the outstanding multiplexing capability of IMS-capable instruments generates vast amounts of data, often containing spatially resolved information for thousands of molecular species in a single experiment (Caprioli *et al.* 1997, Alexandrov 2012, Spraggins *et al.* 2019). The volume and high dimensionality of IMS data present significant challenges in data processing, analysis, and interpretation (Alexandrov 2012). One of the primary challenges is data reduction, as raw IMS datasets typically consist of hundreds of thousands to millions of spatial locations, i.e. pixels, each associated with a mass spectrum containing hundreds of thousands of ion intensities. These datasets contain a mixture of high- and low-intensity peaks as well as features with varying signal-to-noise ratios (SNRs). Managing such large datasets requires effective data reduction techniques that extract meaningful information while minimizing computational burden and storage demands (Verbeeck *et al.* 2020).

Current data reduction in IMS can be broadly categorized into acquisition-time and post-acquisition approaches (see Supplementary Materials for a more elaborate overview). Acquisition-time methods reduce data during collection, typically resulting in a sparse representation rather than describing spectra in the full mass domain. Post-acquisition methods, often user-controlled, include peak picking, spectral integration, and spatial cropping (Monchamp *et al.* 2007, Alexandrov 2012, Anderson *et al.* 2016). These methods aim to further convert IMS data into more manageable representations (Alexandrov 2012, Verbeeck *et al.* 2020). However, methods like peak picking can miss low-intensity, low *SNR* peaks, and near-isobars, potentially introducing bias. Recent efforts have improved peak-picking accuracy (González-Fernández *et al.* 2023), but challenges remain in handling near-isobaric species and low-intensity peaks.

Here, we introduce a novel data model for IMS measurements that addresses missing values through sparse-format-aware low-rank matrix approximation. This approach offers an alternative to traditional data reduction methods for IMS, mitigating selection bias and minimizing information loss early in the analysis pipeline by preserving full spectrum information rather than only selective sub-windows of the measured mass range. Additionally, to handle the computational demands and large memory requirements of modern IMS datasets, we explore the use of randomization strategies to optimize low-rank factorization methods and implement obtaining such a representation of an IMS dataset.

## 2 Materials and methods

Consider a MALDI-TOF IMS dataset *M* ($\in R^{m\times n}$), where *m* is the number of pixels/spectra, *n* is the number of *m/z*-bins recorded by the instrument, and $M_{ij}$ is the ion intensity value associated with the *i*-th pixel and *j*-th *m/z*-bin. This dataset consists of real-valued ion intensities, where a row of *M* is a spectrum associated with a specific spatial location in the tissue and where a column of *M* is a particular *m/z*-bin considered across all pixels. The latter can be reconstructed into a so-called ion image, reporting the spatial distribution and abundance of a specific *m/z*-bin’s intensities. For some IMS experiments, the intensity values $M_{ij}$ are clipped by the instrument (e.g. explicitly by acquisition-time data reduction or implicitly by the instrument’s limit-of-detection), effectively not reporting intensity values below a certain relative ion count *k*. Ion intensity clipping is sometimes expressly performed to induce a sparse regime on the recorded signals, often to save disk space. (In this context, sparsity refers to the number of non-zero values in measurements, i.e. high sparsity implies many zero values. A sparse regime implies that measurements contain many zero values.) Regardless of the reason for clipping to occur, we want to explicitly deal with the missing values introduced by it. Therefore, we propose to model clipping as a function $f:R^{m\times n}\to R^{m\times n}$,


(1a) $$\begin{array}{c} f(M)={\left[f_{ij}(M)\right]}_{m\times n} \end{array}$$

where $f_{ij}(M)$ is defined for each entry (i,j) as:


(1b) $$f_{ij}(M)=\{\begin{array}{cc} M_{ij} & \text{if}\;M_{ij}\geq k\\ 0 & \text{if}\;M_{ij}<k \end{array}$$

for i∈[1,m] and j∈[1,n]. The resulting (sparsified) dataset f(M)  $\in R^{m\times n}$ can be stored in a sparse matrix format, a data structure that only explicitly stores non-zero values and their locations in the matrix, leaving zero values to be implicitly represented without consuming memory. Most post-acquisition data reduction methods ignore the non-linear operator, f(·) (see Supplementary Materials). By applying a sampling operator $P_{\Omega }(\cdot )$ to *M*, essentially a relaxation for f(M), we acknowledge that there are missing values in *M* and avoid the assumption that those missing values are necessarily zeroes when modeling. The sampling operator also avoids that those missing values (potentially) negatively impact the model. Specifically (Candes and Plan 2010), $P_{\Omega }:R^{m\times n}\to R^{m\times n}$ is defined as


(2) $${\left[P_{\Omega }(M)\right]}_{ij}=\{\begin{array}{cc} M_{ij} & \text{if}\;(i,j)\in \Omega \\ 0 & \text{if}\;(i,j)\notin \Omega \end{array}$$

for i∈[1,m], j∈[1,n], and where Ω is the set of indices corresponding to the (known, reported) sampled values, as obtained from the instrument and after any acquisition-time data reduction. We denote (i,j)∈Ω as the set $\Omega_{c}$, making Ω and $\Omega_{c}$ complementary subsets of all entries in *M*. We can formulate the modeling of an IMS dataset *M* implicitly as a missing value problem, wherein a low-rank matrix approximation *X* of *M* is sought in the presence of missing data. The *M*-approximating matrix *X* can be considered an underlying model for the observed measurements in *M*, and the rank of *X* denotes the dimension of the subspace containing the approximating matrix (see Supplementary Materials).

Since the proposed rank-optimization problem is non-convex, NP-hard, and thus difficult to calculate, we instead solve a convex relaxation of the problem [see Supplementary Equation (S1)] using the singular value thresholding (SVT) algorithm (Cai *et al.* 2010, Candes and Plan 2010):


(3) $$\begin{array}{c} \underset{X}{\text{minimize}}\;||X|{|}_{*},\\ \text{subject}\;\text{to}\;\;P_{\Omega }(M)=P_{\Omega }(X). \end{array}$$

This program is shown to exactly recover the solution of the original problem [see Supplementary Equation (S1)] under specific conditions, e.g. incoherence of bases and sampling distribution (Candes and Recht 2012). As proving a condition’s validity is considered to be as hard as solving the original problem [see Supplementary Equation (S1)], the conditions cannot be verified. Thus, we will assume that conditions are met. Nevertheless, we will discuss the sampling distribution assumption in the Case Study section of this article, as it is closely intertwined with the clipping mechanism during acquisition-time data reduction.

The SVT’s advantage lies in utilizing matrices in sparse and low-rank format without requiring dense memory storage, crucial for large IMS datasets. However, the singular value decomposition’s (SVD’s) time complexity (Dongarra *et al.* 2018) remains a bottleneck for MALDI-TOF IMS datasets, leading to the adoption of a divide-factor-conquer (DFC) approach to address this issue (see Supplementary Materials). Besides the sparse-format-aware SVT approach and its SVD-related modification to calculate our low-rank approximation of IMS data, we also explore a second method that has a similar solving program as in Equation (3), but it relaxes the equality into an inequality constraint:


(4) $$\begin{array}{c} \underset{X}{\text{minimize}}\;||X|{|}_{*},\\ \text{subject}\;\text{to}\;||P_{\Omega }(M)-P_{\Omega }(X)|{|}_{F}^{2}\leq \sigma . \end{array}$$

The latter enables the use of a fixed point continuation (FPC) algorithm for solving instead (Candes and Plan 2010, Ma *et al.* 2011). This second program accounts, in addition to missing values, for low-intensity dense noise (e.g. Gaussian noise) in the measurements, which is also inherently present in IMS data. The disadvantage of this algorithm is that it requires a dense-format matrix of similar size as *M* to be stored in memory (for the MALDI-TOF IMS dataset in this article, this amounts to 1649.769 GB). As such, it is clear that whether SVT or FPC is the better choice for solving these optimization problems depends on the resources available and the needs of the subsequent analysis. Finally, note that both SVT and FPC’s practical implementations have a δ and τ parameter that arise as part of their solving algorithms. These hyperparameters require *a priori* setting (or optimization). We specify their setting for each experiment in the Supplementary Materials.

Furthermore, to deal with both SVD complexity and memory load, we make use of the DFC approach (Mackey *et al.* 2015). It consists of three steps and provides a framework that we can apply to both the SVT and FPC algorithms for obtaining an approximation $\bar{X}$ of matrix *M* with completion for the complete dataset *M*. Its specifics are provided in the Supplementary Materials.

## 3 Case studies

**Figure 1.**
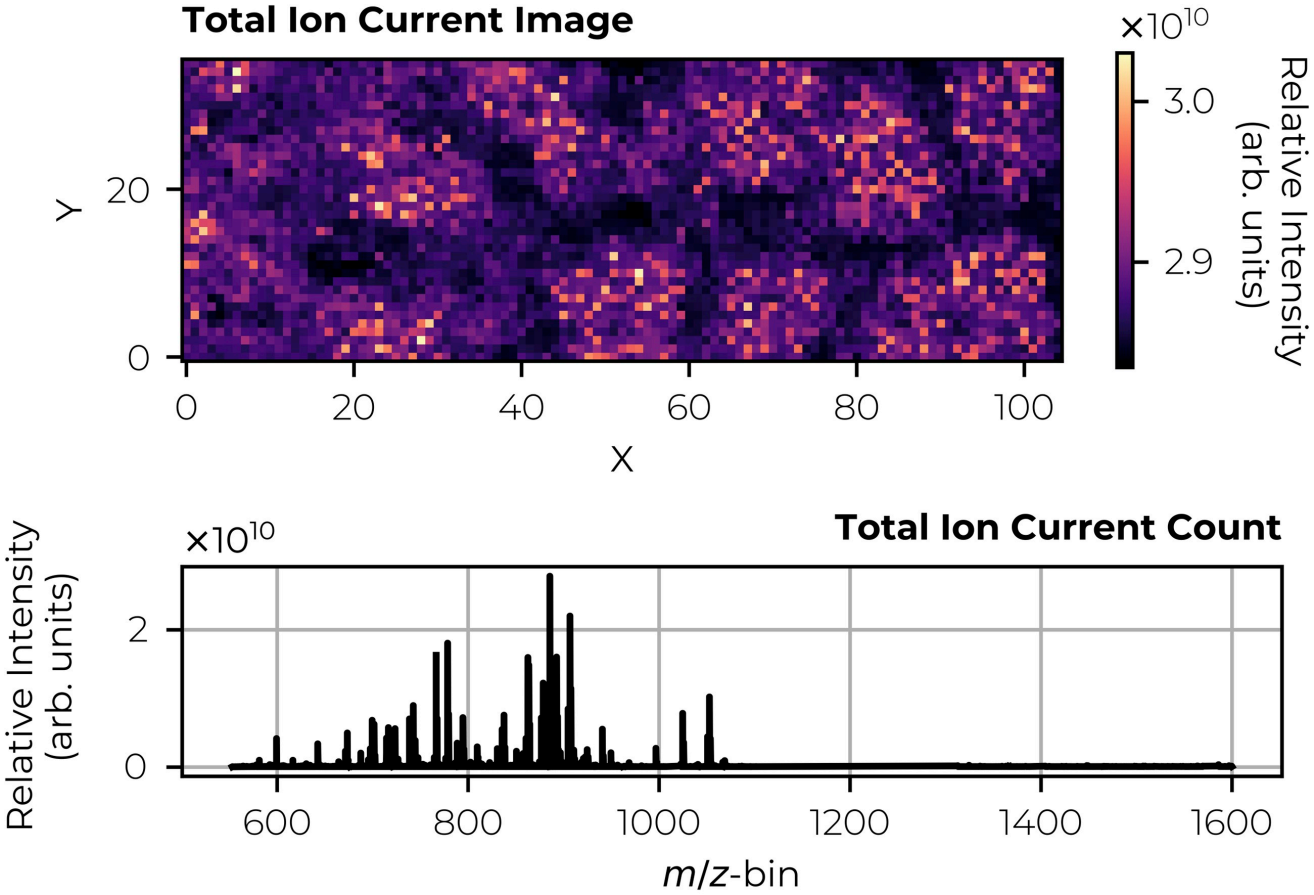
MALDI FT-ICR IMS measurement of human kidney tissue. The experiment was conducted using a 15T Bruker MALDI FT-ICR mass spectrometer (Bruker Daltonics, Billerica, MA, USA) with 50-µm pixel size, covering the *m/z* range from 552 to 1600 in negative ionization mode. For further sample preparation specifics, see the Supplementary Materials. The raw data were exported to a custom file format and normalized using 5–95%-TIC. The dataset contains 3780 spectra, each consisting of 1 372 421 *m/z*-bins. For further data preprocessing specifics, see the Supplementary Materials. The top panel shows the spatial distribution, represented as a total ion current image (i.e. the summation over the normalized spectral axis). The bottom panel displays the summed mass spectrum.

**Figure 2.**
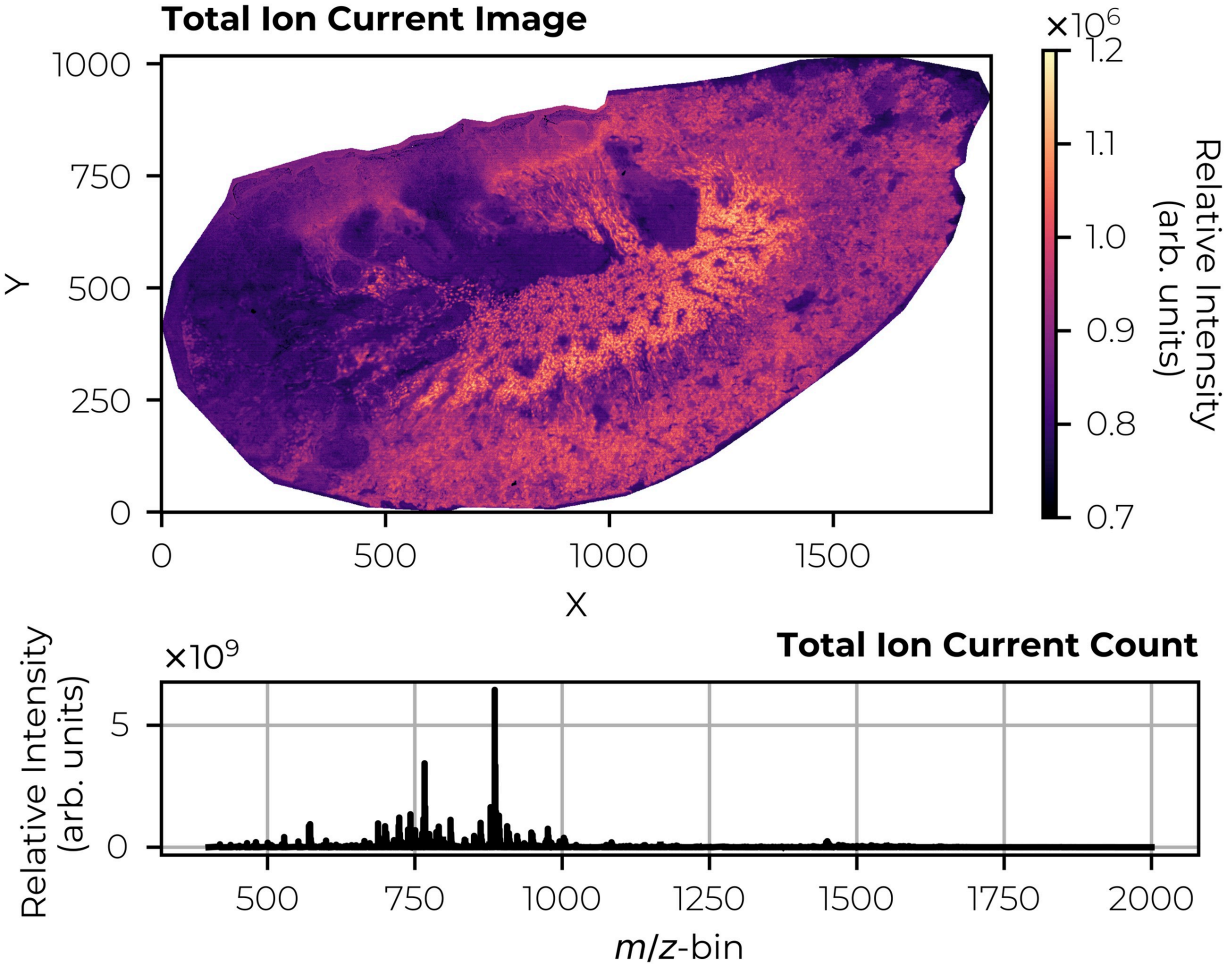
MALDI qTOF IMS measurement of *Staphylococcus aureus*-infected mouse kidney tissue. The infection-induced abscesses are visible as dark areas in the total ion current image. The experiment was performed using a Bruker timsToF Flex mass spectrometer (Bruker Daltonics, Billerica, MA, USA) with 5-µm pixel size, covering a *m/z* range from 400 to 2000 in negative ionization mode. For further sample preparation specifics, see the Supplementary Materials. The raw data were exported to a custom file format and normalized using 5–95%-TIC. The dataset contains 1 320 876 spectra, each consisting of 312 249 *m/z*-bins. For further data preprocessing specifics, see the Supplementary Materials. The top panel shows the spatial distribution of the total ion current image. The bottom panel displays the summed spectrum.

We demonstrate the method’s applicability across different but relatively common instrumental platforms for IMS. Although these case studies focus on specific datasets, the algorithms have not been customized to any particular instrumental setup or IMS dataset type, suggesting that the basic approach could be useful in other types of IMS experiments as well. In a first case study, we establish that an IMS dataset representation using a low-rank matrix factorization approach can outperform an equally small IMS dataset representation using traditional peak picking in a no-missing value case. We demonstrate this on Fourier-transform ion cyclotron resonance (FT-ICR) IMS data (Fig. 1). In the second case study, we investigate the reconstruction error (i.e. on sampled/known values, ∈Ω), the imputation error (i.e. on missing values, $\in \Omega_{c}$) and the global error (i.e. on all entries, $\in (\Omega \cup \Omega_{c})$) on the same FT-ICR IMS dataset as in the first case study (Fig. 1). To mimic missing entries in the FT-ICR data, we implement two sampling schemes. The goal of the third case study is to evaluate the methodology, specifically, the SVT and FPC algorithms with the DFC approach, directly on TOF IMS data that inherently include missing values (Fig. 2). This dataset consists of 312 249 *m/z*-bins for 1 320 876 spectra (1.65 TB in dense matrix format). The evaluation is both quantitative, using an error score and compression factor, and qualitative, with a focus on visualizing advantages and limitations that are relevant to analytical chemists. The data preprocessing can be found in the Supplementary Materials.

### 3.1 Case study 1: low-rank matrix factorization outperforms traditional peak picking

We first demonstrate that, in addition to retaining full spectrum information, a low-rank matrix approximation can achieve a lower reconstruction/global error compared to peak picking in a no-missing value case. Having established this baseline, we can then expand our problem setting with missing values in the second case study.

**Figure 3.**
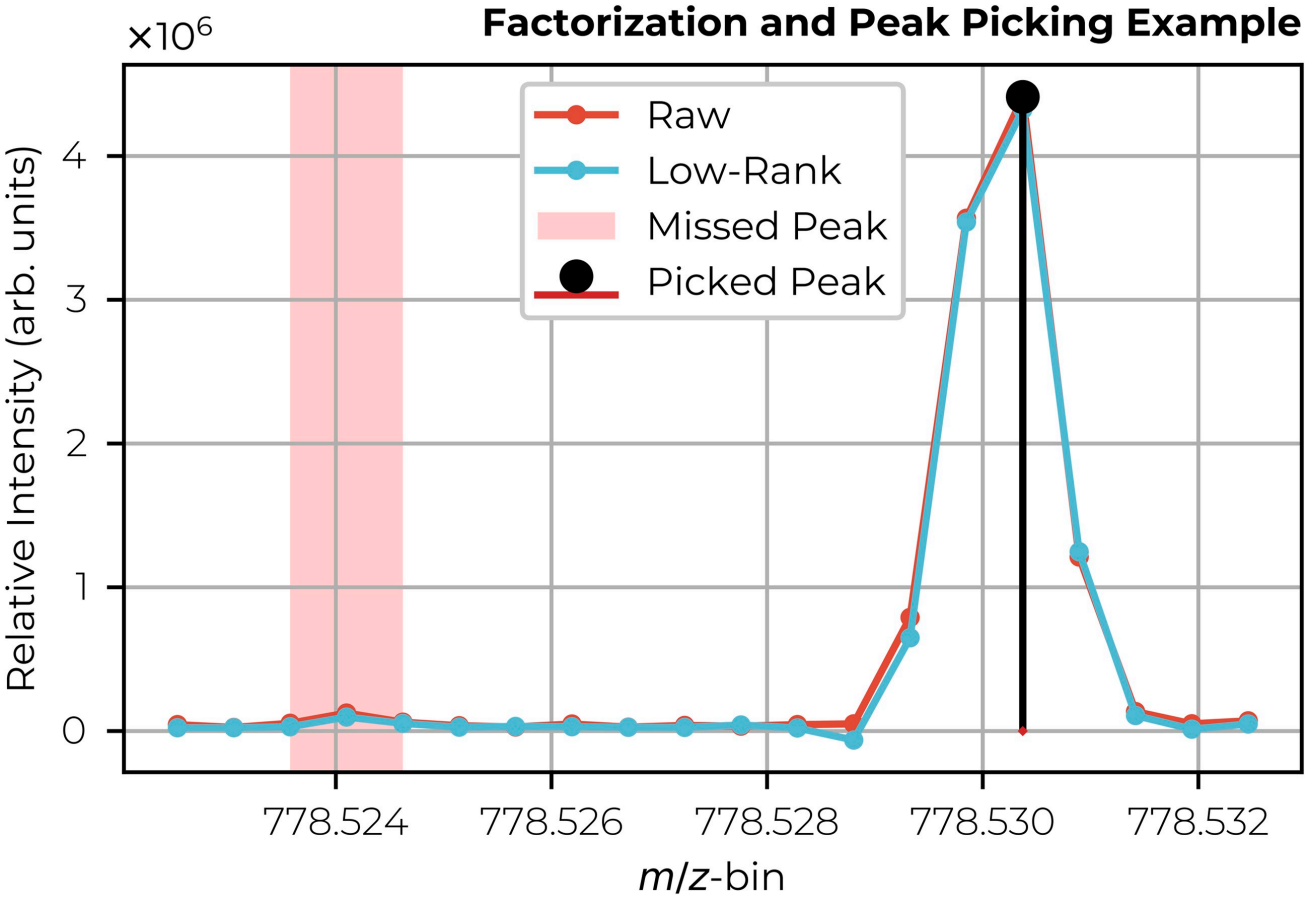
MALDI FT-ICR spectrum of a particular pixel showing the raw, low-rank, and peak-picked data in a particular *m/z*-window of the mass spectrum. The area, highlighted around *m/z* 778.524, shows a low-abundant peak missed by the peak-picking representation, yet accurately captured and reconstructed by the low-rank factorization representation (see Supplementary Materials for the corresponding raw and imputed ion images). This is achieved at identical compression ratios for both representations. If low-abundant peaks are of interest, factorization-based representations are probably better suited to reduce the dimensionality of IMS datasets.

The best rank-*k* approximation with respect to the Frobenius norm (a measure we will use throughout this article) is given by the truncated SVD (Eckart and Young 1936, Mirsky 1960). Since peak picking can be viewed as a form of (low-rank) matrix approximation by selecting specific columns from a dataset (with a column representing a selected peak), we can assert that peak picking is, at best, as effective as the truncated SVD. In Table 1, we observe a 39.1% difference in reconstruction error (which is equal to the global error in the absence of missing values) between the truncated SVD (factorization) and peak picking (100 peaks, see Supplementary Materials for extended numbers of picked peaks). Even if we compare the reconstruction error for a similar data footprint, this still amounts to a difference of 36.8%. While a reconstruction score can be a rather abstract form of capturing full spectrum information content, we highlight in Fig. 3 a concrete difference between the raw IMS data, a low-rank representation (rank-100), and a conventional peak-picked representation. This example demonstrates that not only is the overall ion intensity profile preserved in the factorization representation but also a low-abundant peak at *m/z* 778.524 is effectively retained, where the peak-picked representation misses this peak entirely. Both the metric used in Table 1 and the example of missing low-abundance peaks in Fig. 3 illustrate that low-rank matrix factorization can outperform traditional peak picking when it comes to IMS dimensionality reduction. However, while SVD is a strong factorization method, it may not always be optimal, e.g. when dealing with missing values in the data.

**Table 1.** Comparison of truncated SVD and peak picking results.^a^

**Method**	Rank	**Reconstruction error** $\frac{||M-X|{|}_{F}}{||M|{|}_{F}}\times 100\%$	Compression factor w.r.t. dense format	**Dense data footprint (**GB**)**
*Raw*	–	0	–	20.7510
*Peak picking (100 peaks)*	100	60.5	13 724	0.0015
*Peak picking (123 peaks)*	123	58.2	11 158	0.0019
Truncated SVD	100	21.4	11 158	0.0019

a The reconstruction error is used throughout this article as a metric to measure how well (full) spectrum information is captured. A larger error implies that more information is lost. Hence, a low error is desired. However, note that an error of 0% is (probably) not desired as the data contains noise and it would be desirable to filter off this noise, leading to a (small) error. From this table, we observe that a factorization approach, the truncated SVD, leads to a substantial decrease in reconstruction error (up to 39.1%) compared to peak picking. The truncated SVD is carried out by truncating an SVD performed by the GESDD routine. Peak picking is performed by matching (i) the rank and (ii) the data footprint, i.e. MBs on disk. The raw data in dense matrix format have a storage footprint of 20.751 GB.

### 3.2 Case study 2: reconstruction and imputation quality when dealing with missing values

Having established that a factorization approach is favorable over peak picking in a no-missing value situation, our factorization approach is now evaluated in a missing value scenario. For this case study, we therefore implement two sampling schemes to mimic missing values in IMS data:

(α) Selects the top 8.9% of intensity values (to establish the in-sampling set Ω, i.e. the known values), with all other (lower) intensity values removed (making up the out-of-sampling set $\Omega_{c}$, i.e. missing values),
(β) Selects 8.9% of entries, not based on intensity but, uniformly at random (in-sampling set Ω, i.e. the known values), with all other values removed (out-of-sampling set $\Omega_{c}$, i.e. missing values).

Scheme α mimics commonly employed IMS acquisition-time data reduction, while scheme β examines discrepancies related to incoherence conditions imposed by most matrix completion algorithms (Candes and Plan 2010, Candes and Recht 2012). The 8.9% sampling rate was chosen for IMS fidelity, matching the real-world TOF IMS dataset sampling rate in the third case study. Table 2 presents error scores for both the SVT and FPC algorithms (without the DFC approach) using sampling scheme α on the FT-ICR IMS dataset. It reports: 

**Table 2.** Comparison of SVT and FPC results, with threshold sampling scheme α.^a^

**Input** *M*	**Reference** $\tilde{M}$	Method	Rank	**Reconstruction error** $\frac{||P_{\Omega }(\tilde{M}-X)|{|}_{F}}{||P_{\Omega }(\tilde{M})|{|}_{F}}\times 100\%$	**Imputation error** $\frac{||P_{\Omega_{c}}(\tilde{M}-X)|{|}_{F}}{||P_{\Omega_{c}}(\tilde{M})|{|}_{F}}\times 100\%$	**Global error** $\frac{||\tilde{M}-X|{|}_{F}}{||\tilde{M}|{|}_{F}}\times 100\%$
*Raw*	*Raw*	*Peak picking*	100	−	−	60.5
Raw	Raw	SVT	100	26.6	66.7	34.7
Raw	Raw	FPC	100	13.7	59.2	25.0
Raw	Low-rank	SVT	100	26.5	51.5	31.4
Raw	Low-rank	FPC	100	6.2	33.2	13.8
Low-rank	Low-rank	SVT	100	4.9	93.1	34.8
Low-rank	Low-rank	FPC	100	2.6	83.4	31.0

a A low reconstruction error is observed for all methods for both raw and low-rank inputs and references, comparable to the no-missing values case. The imputation error is more substantial. However, since these consist mostly of low-intensity values (caused by the clipping operator), their impact is small on the global error. For SVT, we set parameters δ=1 and $\tau =10^{-3}$ and for FPC, we set δ=1.4 and $\tau =10^{-3}$ (see the Supplementary Materials). Peak picking is performed by picking the 100 highest peaks of the total ion current count of the raw data. For SVT with raw input data, we obtain a 171 rank solution, and for FPC, a 271 rank solution. For SVT with low-rank input data, we obtain a 131 rank solution, and for FPC, a 111 rank solution. We truncate all solutions to a rank of 100 for fair comparison. The SVT took on average 64.74 min to converge, while the FPC algorithm took on average 41.89 min.

- Reconstruction error: modeling error for known entries.
- Imputation error: modeling error for missing values.
- Global error: modeling error for both known and missing entries.

Error scores were calculated with respect to both raw data and its low-rank approximation. Therefore, the input matrix is defined as the matrix used as input to our algorithms [Equations (3 and 4)]. The reference matrix is defined as the matrix used as reference in the error scores. We considered two types of input (*M*) and reference ($\tilde{M}$) matrices:

- Raw: A dataset with missing values sampled directly from the raw data (thus including high-rank noise variation),
- Low-rank: A dataset with missing values sampled from a low-rank version of raw data, obtained through truncated SVD with rank 100.

Using the low-rank approximation as input (*M*) and reference ($\tilde{M}$) ensures that the low-rank conditions imposed by SVT and FPC are met, reducing unwanted (often noisy) variation from impacting the evaluation process.

Finally, note that the presented results stem from single experiments influenced by various factors (e.g. tissue type, sample preparation, detector type, raw data structure, noise levels, algorithmic parameters). Consequently, they are only evaluated relative to each other.

#### 3.2.1 Reconstruction, imputation, and global error

As shown in Table 2, both methods exhibit relatively low *reconstruction error* for non-missing values (between 2.6% and 26.6%) across all input and reference matrices. This is comparable/a slight improvement with respect to the results found in the first case study. Generally, FPC outperforms SVT on the raw input matrix, which is expected due to FPC’s ability to filter out small dense noise. On the other hand, both methods show only moderate performance on *imputation error* for missing values (between 33.2% and 93.1%) across all input and reference combinations, with FPC showing a slight advantage. This trend, along with similar results from the uniform sampling scheme β (see the Supplementary Materials), suggests that while a low-rank factorization representation does a great job for capturing non-missing value entries, its performance as a predictor for missing values is limited, and further investigation is needed to better understand the underlying causes.

Interestingly, contrary to expectations, the uniform sampling scheme β does not outperform the threshold-based sampling α, despite its closer alignment with incoherence conditions. This highlights the need to carefully consider the implications of different sampling strategies.

**Figure 4.**
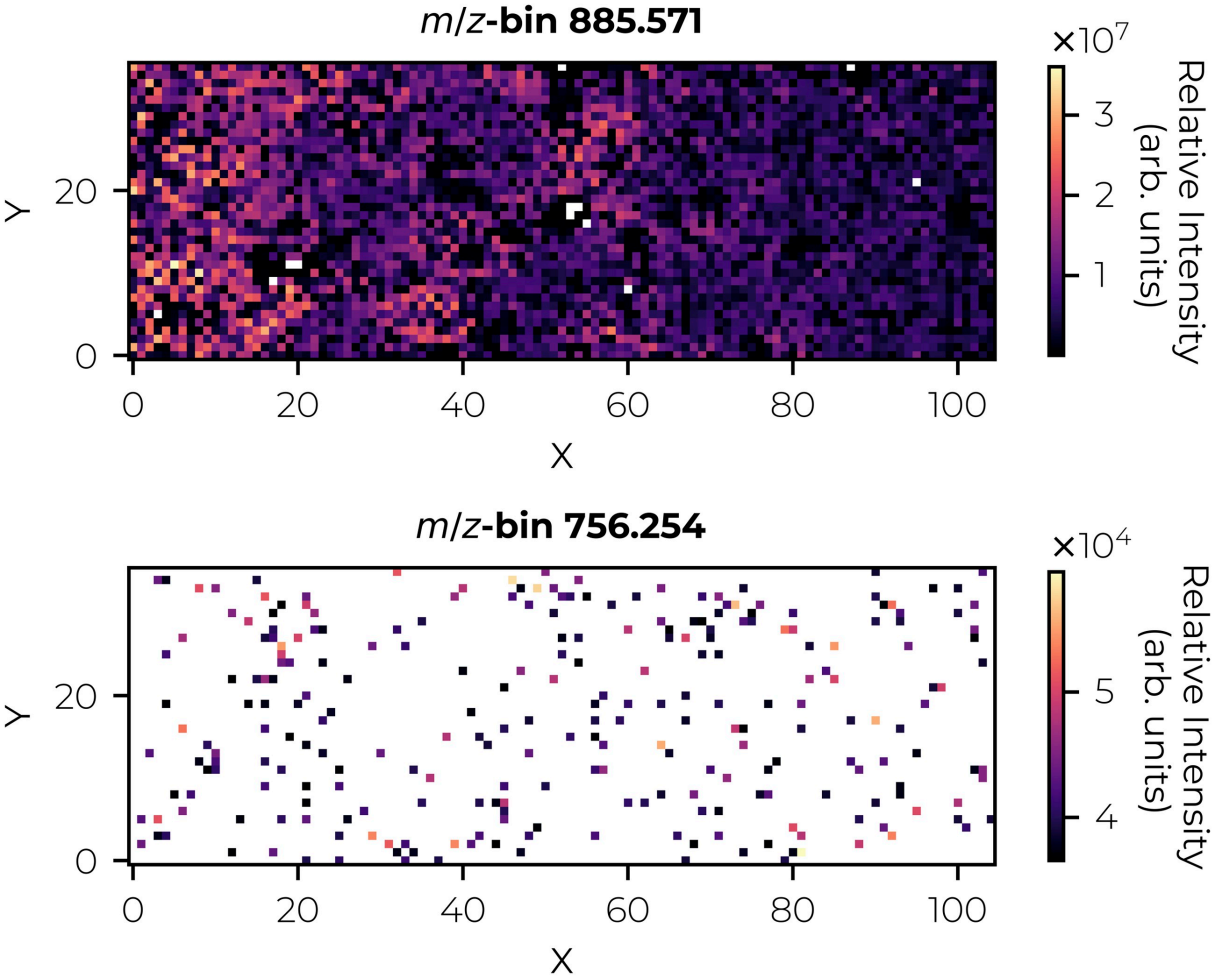
MALDI FT-ICR ion images from a single *m/z*-bin of the low-rank input matrix with threshold sampling scheme α. The top image depicts an *m/z*-bin with high intensity and thus, with the scheme α, a low number of missing values. The bottom image depicts an *m/z*-bin with low intensity and thus, with scheme α, a high number of missing values.

Threshold sampling scheme α removes low-intensity entries, primarily associated with noise. Hence, it requires the imputation of noisy features by a low-rank model. However, this scheme generally fails to satisfy incoherence conditions, leading to poor imputation error in general. The poor performance could, for example, be caused by the spatial correlation of low-abundance values, which is particularly evident in specific *m/z*-bins and distinct (positive) spatial areas across the tissue. As illustrated in Fig. 4, under an intensity-magnitude driven sampling scheme α, a high-intensity *m/z*-bin at 885.571 is missing only a few values (white entries), while a low-intensity *m/z*-bin at 756.254 can be missing many values.

In contrast, a uniform sampling scheme β is expected to perform better because it more closely aligns with incoherence conditions and treats all ion species the same. However, we observe worse reconstruction and imputation errors compared to scheme α. This is probably related to (i) the low 8.9% sampling rate (i.e. 91.1% of all intensity entries are missing in this dataset and there is relatively little signal to model with), and (ii) the predominant number of low signal-to-noise *m/z*-bins in the raw data. Consequently, uniform sampling leads to a significant loss in high-valued, “informative” entries. We expect that the imputation error for sampling procedure α and both reconstruction and imputation error for sampling procedure β could benefit from advanced feature scaling. Additionally, introducing chemical noise (e.g. speckle noise) into our data before applying the sampling scheme could cause high-intensity values to become missing, albeit at a lower rate, which may further reduce the imputation error.

Nevertheless, both methods achieve a *global error* that is 25.7–46.7% lower than that of peak picking, which has a global error of 60.5%, representing a substantial improvement in terms of full spectrum information, even in the presence of missing values. Moreover, note that the imputation error does not significantly influence the global error score since low-intensity features (at least 100-fold smaller than the base peak) contribute less to the global error—due to the properties of the Frobenius norm and because the sampling scheme α retains high-intensity values (see Supplementary Materials for ion image examples). Overall, this result implies that full spectrum information is better captured by the proposed low-rank factorization methodology than by peak picking, both when missing values are present as well as when they are absent (see Supplementary Materials for *k*-means clustering comparison).

### 3.3 Case study 3: advantages and disadvantages of low-rank matrix completion for missing value TOF IMS data

In this case study, we forgo synthetically generated missing values, for which the ground truth is known, and apply our approach on IMS data with intrinsic missing values.

#### 3.3.1 Reconstruction error and compression factor

The performance metrics for this third case study, including the reconstruction error and compression factors, are summarized in Table 3. They highlight that both SVT and FPC exhibit comparable performance. While a reconstruction error of ∼20% might seem substantial at first, it actually represents a 30% reduction in information loss compared to traditional peak picking, all while maintaining the same data footprint and enabling full profile analysis in downstream workflows. Furthermore, all methods demonstrate high compression factors, with the IMS representation’s footprint being ∼2500 times smaller compared to a dense matrix format and 600 times smaller than a sparse matrix format, comparable to those achieved by peak picking.

**Table 3.** Comparison of SVT and FPC results, with randomized projection divide-factor-conquer approach.^a^

Method	Rank	**Reconstruction error** $\frac{||P_{\Omega }(\tilde{M}-X)|{|}_{F}}{||P_{\Omega }(\tilde{M})|{|}_{F}}\times 100\%$	Compression factor w.r.t. dense format	Compression factor w.r.t. sparse format	**Dense data footprint (**GB**)**
*Raw*	–	0	–	–	1649.769
*Peak picking (123 peaks)*	–	52.0	2525	642	0.653
SVT	100	21.6	2525	642	0.653
*Raw*	–	0	–	–	1649.769
*Peak picking (129 peaks)*	–	51.4	2405	611	0.686
FPC	105	23.6	2405	611	0.686

a A substantial improvement for the reconstruction error is observed for both SVT and FPC in comparison to peak picking, while compression factors and data footprint are equal. For SVT, we set parameters δ=1.7 and τ=.5 and for FPC, we set δ=1 and $\tau =1.5\times 10^{-2}$ (see the Supplementary Materials). Peak picking matches the data footprint, i.e. MBs on disk. The raw data in dense matrix format have a storage footprint of 1649.769 GB, and storing it in a sparse matrix format (e.g. compressed sparse column) amounts to 279.648 GB. The full process of dividing, factoring, and combining took around 12 h for the SVT and around 8 h for the FPC.

#### 3.3.2 Spectral error distribution and biological interpretation

We further investigate the distribution of reconstruction errors for individual spectra, referred to as the spectral error score (see Supplementary Fig. S14a and c). This score is calculated both for

- the 100 *m/z*-bins with the largest total ion current count across the dataset; and
- the largest 100 *m/z*-bins per spectrum, i.e. the top peaks in each individual spectrum.

The distributions of spectral error scores reveal patterns that correlate with biology for both SVT and FPC methods under both scoring criteria (*a* and *b*). Interestingly, the spectral error is slightly lower for the largest individual spectrum peaks (*b*), as the top dataset-wide peaks (*a*) might not be present in every spectrum. The error distributions appear to be a mixture of two Gaussian-like distributions with different means and standard deviations. Spatial reconstruction of these distributions (Supplementary Fig. S14b and d) reveals distinct tissue regions that may correlate with the total ion current count (Fig. 2). Moreover, no clear relationship is observed between these distributions and the number of non-zero values per spectrum (see Supplementary Fig. S18). This suggests significant heterogeneity in molecular distributions within the tissue, rather than issues related to incoherence, might be influencing the reconstruction quality, especially in *Staphylococcus aureus*-infected regions.

#### 3.3.3 Methodological effects on reconstructed ion images and spectra

Although high-intensity ion images and peaks are recovered well (see reconstruction error and spectral error score, and the Supplementary Materials for ion image examples), distortions may occur in the reconstructed spectra and individual ion images of very low intensity (Supplementary Fig. S15). Commonly observed distortions included (i) small peak shifts, i.e. shifting of peak distribution along the *m/z* axis, (ii) peak widening, i.e. smearing peaks over larger *m/z* ranges, and (iii) peak prediction, i.e. imputation of peaks not present in the raw spectrum, but predicted on the basis of dataset-wide observed patterns. It should be noted that these effects are not necessarily incorrect, that they may arise from genuine corrections for small non-linear misalignments due to instrumentation or noise, or from other instrumental artifacts.

Notably, these distortions are more prominent in low-intensity peaks (mostly around and below $10^{3}$ relative intensity in peak height), which is consistent with the optimization process focused on minimizing the Frobenius norm. This introduces (iv) a recovery bias that favors better reconstruction of high-intensity peaks, as also observed in this TOF IMS dataset. From a spatial perspective, caution is warranted when interpreting very sparse ion images as biologically meaningful. For example, the predicted ion image of *m/z* 1284.10, is based on only very few measurements (see raw ion image of *m/z* 1284.10, Supplementary Fig. S15). These low-abundant species-centric effects, whether desired or undesired, can potentially be mitigated in the future through advanced feature scaling and an improved model. They should also always be considered within the context of peak picking approaches, which often leave no record of low-abundant species to begin with.

#### 3.3.4 Preservation of near-isobaric species

Near-isobaric species, molecular species with nearly identical mass-to-charge ratios but different chemical compositions, pose significant challenges for accurate peak detection. These species are often overlooked in peak picking due to their low intensity relative to dominant peaks, or may be incorrectly integrated as a single species. In Supplementary Fig. S16, we present an example of such a near-isobaric species, at approximately *m/z* 725.53 (blue area), located close to a dominant species at *m/z* 725.51 (orange area). Due to their proximity, near-isobaric species are frequently neglected. Integrating the orange and blue areas separately, reveals different spatial molecular distributions, indicating that these *m/z*-ranges correspond to distinct molecular species. When applying peak picking, the best-case scenario consists of integrating the orange area and neglecting the blue area, potentially missing the near-isobaric species. In the worst-case scenario, both areas are integrated as one, and the dominant peak’s intensity overshadows that of the near-isobaric species, leading to the loss of unique spatial information. In both cases, the unique near-isobaric information is lost. However, our methods successfully preserve this information by approximating the full spectrum without requiring prior specification of their positions on the *m/z*-axis. This ensures that near-isobaric species are more accurately captured and represented, maintaining the unique spatial and molecular information they provide. Preserving near-isobaric species is an important factor in improving analysis specificity. Specificity on an instrumental and/or (bio)chemical level is an important driver in IMS (Spraggins *et al.* 2019), being able to maintain it in the analysis is thus of utter importance.

#### 3.3.5 Retention of lower-intensity ion species and bias mitigation

Our approach effectively mitigates bias by retaining lower-intensity ion species that are commonly disregarded by peak picking, especially when only the largest peaks are retained. We identified several *m/z*-bins representing peaks corresponding to biologically relevant lipids and adducts, which were preserved in our analysis despite their low intensity (Supplementary Fig. S17). The specific *m/z* values include:

- The lipid LPE 18:1 at *m/z* 478.29 (confirmed by liquid chromatography mass spectrometry),
- A 4-(dimethylamino)cinnamic acid (see Supplementary Materials) adduct of PE O-(36:3) at *m/z* 917.54,
- [CL(77:2)+Na-2H]- at *m/z* 1552.12.

These *m/z*-bins are not isotopic peaks and thus provide unique information about species abundant in different spatial regions in the tissue. These examples are only a few from a large group of peaks (1000+) that are preserved for this TOF IMS dataset. Retaining low SNR signals enhances the detection of species in downstream analyses, reducing confirmation bias by reporting on nearly all instrument-detected peaks rather than narrowing the analysis pre-maturely to a set of high-abundant species. Retention of lower-intensity ion species in the computational representation and analysis is an important factor in maintaining sensitivity throughout the chain from sample preparation to instrument to computational analysis to biological insight.

## 4 Conclusions

This article explored the application of matrix factorization algorithms on IMS data, focusing on the goal of dimensionality and data footprint reduction, addressing the issue of missing values, and evaluating both quantitative and qualitative outcomes. For a no-missing value case, a low-rank factorization-based representation of IMS data improved the reconstruction error by 39.1% over peak picking while concurrently maintaining a full spectrum profile for all spectra in the dataset. In the missing value case, we achieved low reconstruction errors for both SVT- and FPC-based approaches, comparable to the no-missing value case. We also highlighted the persistent challenge of reducing imputation errors, which could potentially be mitigated through advanced feature scaling that accounts for the specific characteristics of IMS data and an improved data model. For the missing value case, we demonstrated a substantial reduction in full spectrum information loss (global error) up to 40% compared to traditional peak picking methods, while achieving compression factors similar to peak picking. Our experiments revealed that matrix completion algorithms offer significant advantages in maintaining sensitivity by preserving lower-SNR signals and mitigating selection bias. At the same time, we demonstrated the preservation of specificity by retention of near-isobaric species in the analysis through our full profile approach. These improvements are expected to enhance downstream analysis by providing a richer, more complete reduced representation of IMS data while also providing dimensionality reduction capabilities comparable to traditional peak picking. The importance of this research lies in the introduction of a framework for IMS data reduction by factorization in an early stage and with awareness of missing values. This framework enables high compression rates, up to 2500-fold compared to dense matrix storage formats and up to 600-fold compared to sparse matrix storage formats, while preserving substantially more full profile information than peak picking. We emphasize the importance of utilizing full spectra in downstream analysis to avoid premature or biased information loss, as often occurs with peak integration or peak picking. However, our methods also have limitations, such as peak shifting and widening, low-intensity peak prediction, and the prediction of very sparse ion images.

Looking forward, future work could focus on exploring on-the-fly low-rank approximation schemes that can be employed during data acquisition to enhance accuracy and reduce computational burden. Additionally, it will be important to incorporate considerations for non-negativity, measurement sparsity, and uncertainty. Addressing issues related to peak shifting, widening, and normalization also emerges as a critical area for further research.

In conclusion, our study shows that low-rank factorization-based representations of IMS data can substantially advance the field by reducing full spectrum information loss by 30–40% compared to traditional peak picking methods. This work highlights the potential of matrix factorization and, in particular, completion algorithms for avoiding premature feature selection and for lifting IMS data analysis to the full profile level.

## Supplementary Material

btaf247_Supplementary_Data

## Data Availability

The data underlying this article are available at SurfDrive, at https://doi.org/10.4121/a6efd47a-b4ec-493e-a742-70e8a369f788. The source code is available at https://github.com/vandeplaslab/full_profile.
