# Supplementary material for: Preserving full spectrum information in imaging mass spectrometry data reduction
Source: Bioinformatics. 2025 May 8;41(5):btaf247. doi: 10.1093/bioinformatics/btaf247 (PMC12119130; doi:10.1093/bioinformatics/btaf247)
Supplement: btaf247_Supplementary_Data [file btaf247_supplementary_data.pdf]

Supplementary Materials
for
Preserving Full Spectrum Information
in Imaging Mass Spectrometry Data Reduction

Roger A.R. Moens, Lukasz G. Migas, Jacqueline M. Van Ardenne,
Eric P. Skaar, Jeffrey M. Spraggins, Raf Van de Plas

1 Introduction: Current Data Reduction

Current data reduction approaches can be broadly classified into acquisition-time and post-acquisition data reduction methods. Acquisition-time data reduction involves reducing data during acquisition, typically resulting in a selective representation rather than describing full spectra. For instance, time-of-flight (TOF) mass spectrometers may retain only ion intensity values above a certain threshold, discarding other measured m/z-bins. These representations, while reduced, are often not well-suited for downstream analysis due to their still relatively large size, unstructured feature selection, and sparse-matrix-format storage, commonly requiring reconstruction to the full mass domain (*i.e.*, intensity values for all m/z-bins) and subsequent data re-reduction. Moreover, this process is usually "hard-coded" into the instrument and not under user control.

Post-acquisition data reduction is user-controlled and includes techniques such as peak picking (*i.e.,* selecting a subset of features or masses), spectral integration (*i.e.,* combining features over small mass ranges), and spatial cropping (*i.e.,* selecting a spatial subset of interesting pixels and spectra) [Alexandrov, 2012, Anderson et al., 2016, Monchamp et al., 2007]. Note that ion intensity integration sometimes already happens at the detector and/or instrument level, for example, to counteract space-charge effects. These post-acquisition methods usually aim to convert full spectrum imaging mass spectrometry (IMS) data, that is IMS data with ion intensity values for each m/z-bin in the measured mass range, into a more manageable representation reporting only the signal features deemed important, often certain peaks, and taking up a reduced memory footprint such that it is practical for subsequent analysis [Verbeeck et al., 2020, Alexandrov, 2012]. However, methods like peak picking can miss or inadequately capture certain peaks in the full spectra. These missed signals often include low-intensity or low signal-to-noise-ratio (SNR) peaks and near-isobars (*i.e.*, peaks with nearly identical m/z but representing different molecular species, and sometimes presenting as 'shoulders' in a peak profile). This is often due to their focus on the abundance of signals rather than considering the structured signal presence or absence across measurements. Furthermore, their selective nature can introduce bias by limiting downstream analysis to a subset of (often more abundant) molecular species rather than considering all measured species. Although recent efforts have been made to improve peak-picking accuracy and robustness [González-Fernández et al., 2023], challenges persist, particularly in handling near-isobaric species and low-intensity peaks.

2 Methods

2.1 Non-Linear Operator

Most post-acquisition data reduction methods ignore the non-linear operator, $f(\cdot)$, and treat the dataset $f(M)$ as if it was M by assuming zero ion intensity for entries where $M_{ij} < k$. This is, for example, common in most peak picking algorithms, as they often require a full spectrum profile with an intensity value for each m/z-bin to determine where peaks are located. This approach may lead to information loss by assuming zero intensity where the abundance was low but not zero, and it can ultimately lead to biased feature subset selection, overemphasizing the importance of medium-to-high-abundant molecular species and underrepresenting or ignoring low-abundant species. In contrast, the methods presented here do not ignore the non-linear clipping operator, but rather seek to take it explicitly into account and avoid some of the assumptions listed above. Concretely, we propose to model the non-linear clipping function and describe it as a sampling operator.

2.2 Low-Rank Approach

Low-rank matrices have several favourable mathematical properties in the context of underdetermined systems of equations and are oftentimes used to describe data from the smallest possible set of basis vectors, *i.e.,* "the simplest representation for the given measurements". We formulate the modeling of IMS data, *i.e.*, the missing value problem, as an optimization problem that seeks to capture the IMS data using as little rank as possible, while concurrently being aware of the sampling operator and thus missing values:

$$\begin{aligned} \underset{X}{\text{minimize}} \quad & \text{rank}(X), \\ \text{subject to} \quad & \mathcal{P}_\Omega(M) = \mathcal{P}_\Omega(X), \end{aligned} \tag{1}$$

where $X \in \mathbb{R}^{m\times n}$ and $\mathcal{P}_\Omega(\cdot) : \mathbb{R}^{m\times n} \to \mathbb{R}^{m\times n}$ can be seen as an orthogonal projection projecting a matrix onto the space of $\mathbb{R}^{m\times n}$ matrices with support Ω.

2.3 Singular Value Thresholding

A strong advantage of the SVT is that during the optimization it utilizes the matrices in either a sparse or low-rank format, and it does not require a dense-format copy to be stored in memory. This is an extremely important aspect of this modeling effort, since many IMS datasets simply do not fit in memory when considered in a dense data format. A downside, however, is the singular

value decomposition (SVD) at the center of each iteration of the optimization. Namely, the time complexity of the SVD, $\mathcal{O}(mn^2)$, becomes a bottleneck at the scale of MALDI-TOF IMS datasets, mainly due to a number of BLAS level-2 operations at the heart of the SVD [Dongarra et al., 2018]. However, different solutions exist to reduce, *e.g.*, [Halko et al., 2011]), or completely remove the SVD, *e.g.*, [Zhou and Tao, 2011]. We opt for a divide-factor-conquer approach [Mackey et al., 2015], as it is theoretically well-studied and acts as a framework that we can adapt for a second method.

2.4 Divide-Factor-Conquer Approach

Furthermore, to deal with both SVD complexity and memory load, we make use of the divide-factor-conquer approach (DFC) [Mackey et al., 2015]. It consists of three steps and provides a framework that we can apply to both the SVT and FPC algorithms for obtaining an approximation $\bar{X}$ of matrix M with completion for the complete dataset M. Its first step consists of dividing M into t different matrices C_e, consisting of a subset of M's columns, sampled uniformly at random:

$$C_e = [\mathcal{P}_\Omega(M)]_{\Theta_e}, \ \forall\ e \in [1, t], \tag{2}$$

where Θ_e consists of a set of column indices of size $l = \frac{n}{t}$ and Θ contains t such sets. We assume for ease that l is an integer. As such, we obtain t matrices $C_e \in \mathbb{R}^{m \times l}$. Note that each C_e also has a particular $\mathcal{P}_\Omega^{\Theta_i}$ associated with it, namely the sampling associated to those columns in Θ_i. In the second step, these subsampled matrices are factorized separately using the matrix completion methods, either SVT or FPC. Hence, t low-rank matrices $\hat{X}_e$ are obtained. The rank of the overall approximation $\bar{X}$ is calculated by taking the median of all matrix ranks of $\hat{X}_e$. This rank is utilized in the final step, which consists of reconstructing the factored solutions $\hat{X}_e$ into a final approximate factorization $\bar{X}$. A standard Gaussian matrix $G \in \mathbb{R}^{m \times (k+p)}$ is constructed, with p as oversampling parameter, generally used to improve the reconstruction [Halko et al., 2011]. Next, a power iteration scheme is implemented as $Y = (\hat{X}\hat{X}^T)^q \hat{X} G$, with q as the number of iterations and $\hat{X}$ consists of re-ordering and stacking all low-rank approximations. Finally, the top k singular values of Y are obtained, *e.g.*, by QR decomposition, to form $Q \in \mathbb{R}^{m \times k}$. The final solution $\bar{X}$ is then obtained by

$$\bar{X} = QQ^\dagger \hat{X}, \tag{3}$$

where $\dagger$ is representing the pseudo-inverse. The advantage of the divide-factor-conquer approach is that conditions that arise in matrix completion methods, as presented earlier, also guarantee strong estimation properties for divide-factor-conquer [Mackey et al., 2015]. We implemented the SVT and FPC algorithms as well as the divide-factor-conquer approach for both in an efficient Python object-oriented toolbox, with an eye towards saving memory where most needed and accelerating calculations where possible.

3 Case Study 1: Low-Rank Matrix Factorization Outperforms Traditional Peak Picking

3.1 Reconstruction Error for Different Numbers of Picked Peaks

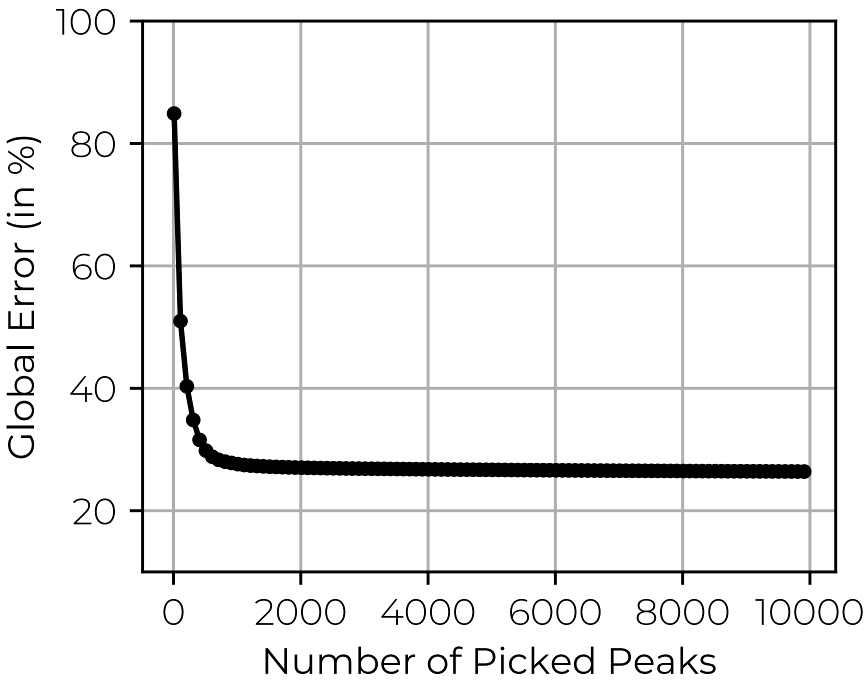

Figure 1: Reconstruction error plot for different numbers of picked peaks.

3.2 Raw and Imputed Ion Images for m/z 778.524

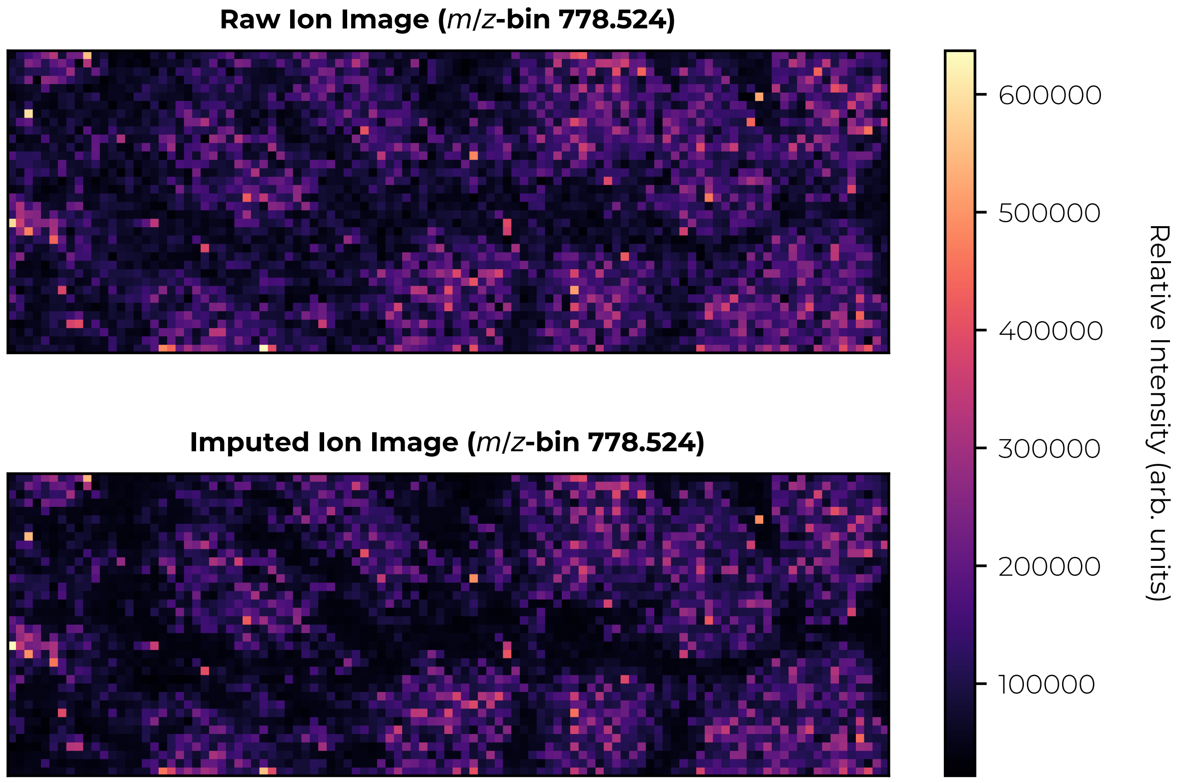

Figure 2: Raw and imputed ion images of m/z-bin 778.524.

4 Case Study 2: Reconstruction and Imputation Quality When Dealing with Missing Values

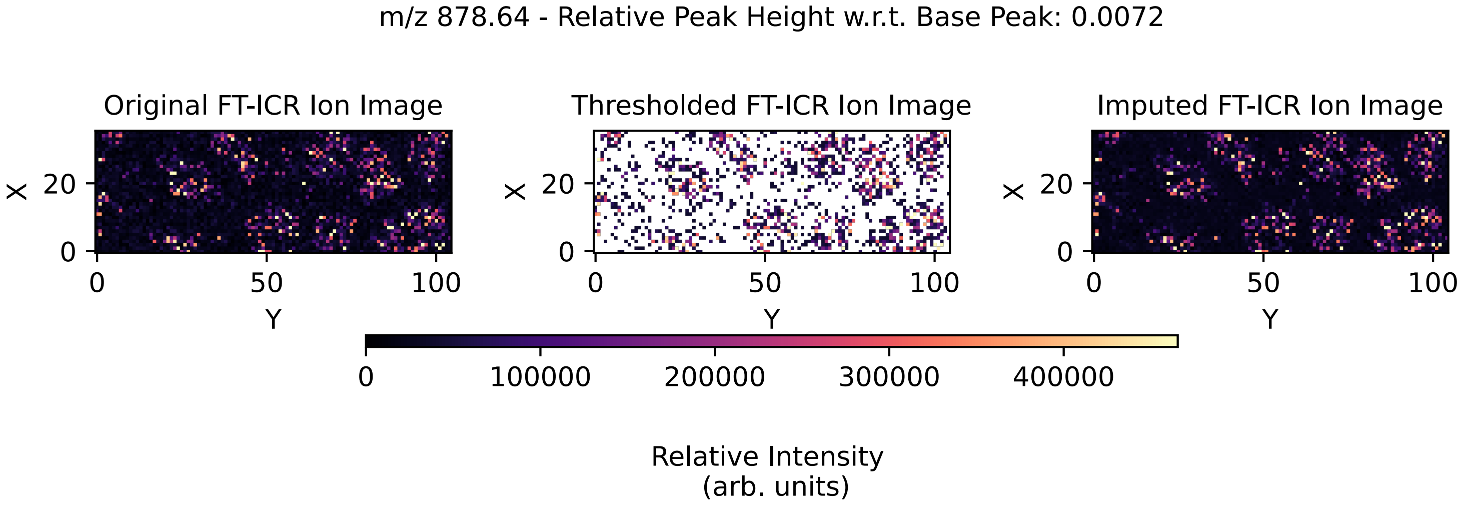

Figure 3: Raw, thresholded and imputed ion image of m/z-bin 878.640. This is a very low-intensity peak w.r.t. the base peak (0.0072 of the base peak height). The right image shows good imputation when visually compared to the original FT-ICR ion image.

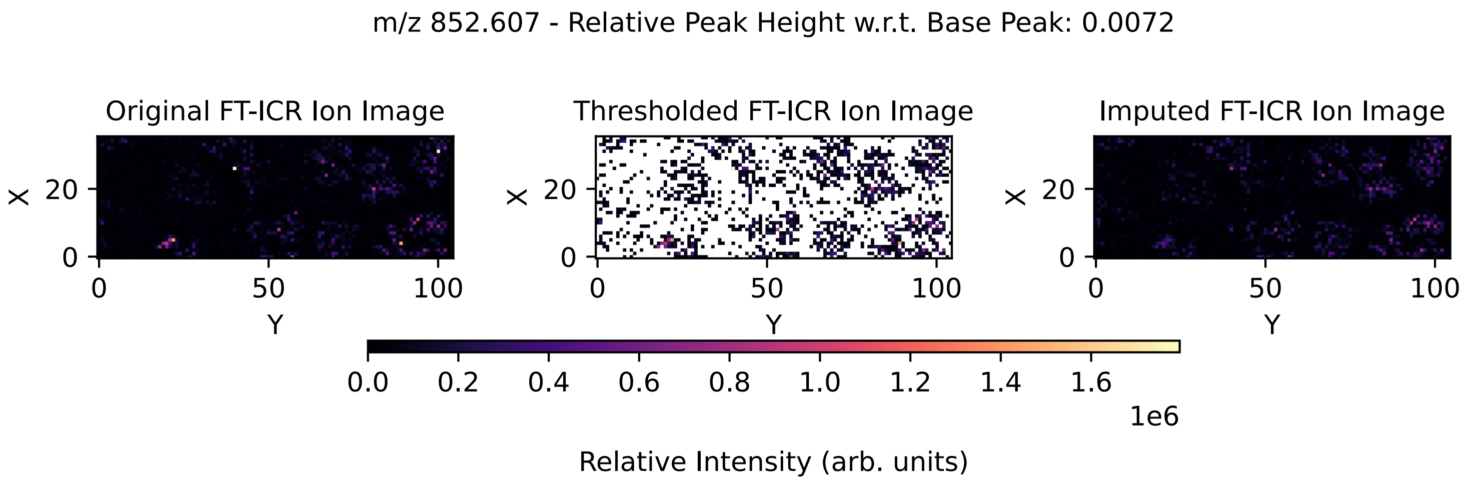

Figure 4: Raw, thresholded and imputed ion image of m/z-bin 852.607. This is a very low-intensity peak w.r.t. the base peak (0.0072 of the base peak height). The right image shows good imputation when visually compared to the original FT-ICR ion image, but it does noticably underestimate high-intensity features.

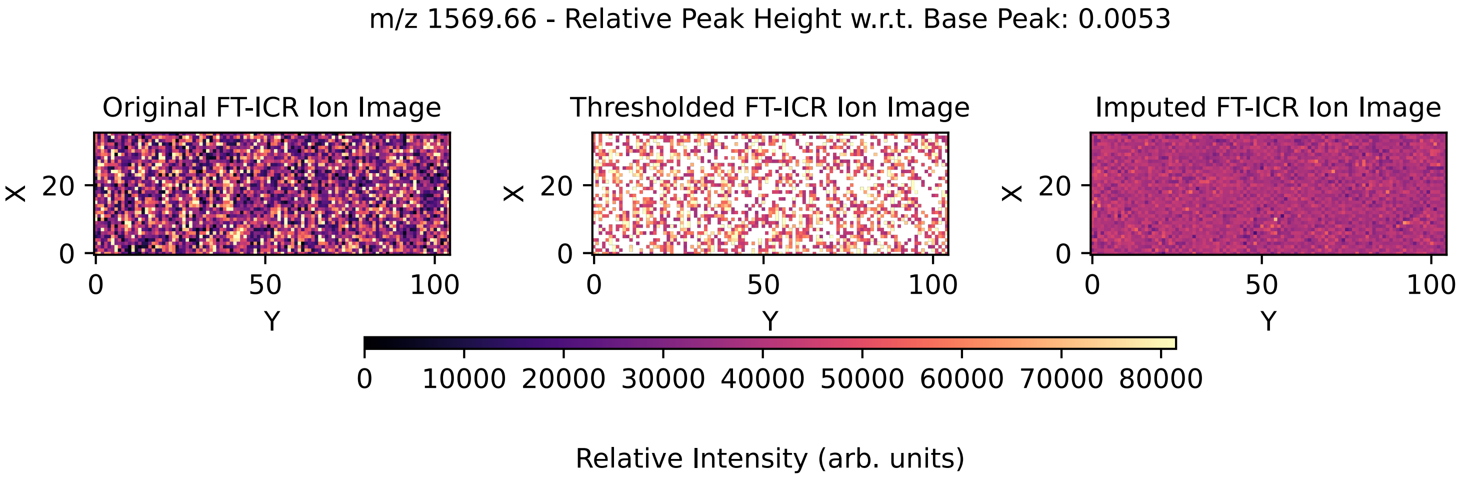

Figure 5: Raw, thresholded and imputed ion image of m/z-bin 1569.66. This is an extremely low-intensity peak w.r.t. the base peak (0.0053 of the base peak height). The right image shows imputation of a noise ion image. This result shows the denoising effect of the approach in action.

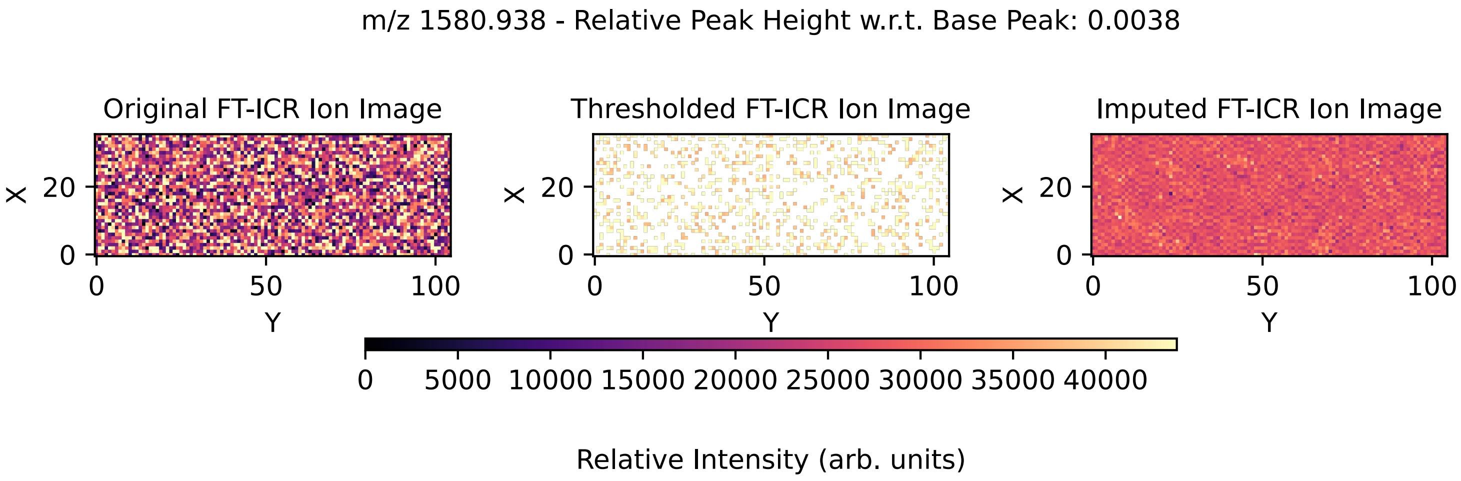

Figure 6: Raw, thresholded and imputed ion image of m/z-bin 1580.938. This is an extremely low-intensity peak w.r.t. the base peak (0.0038 of the base peak height). The right image shows imputation of a noise ion image. This result shows the denoising effect of the approach in action.

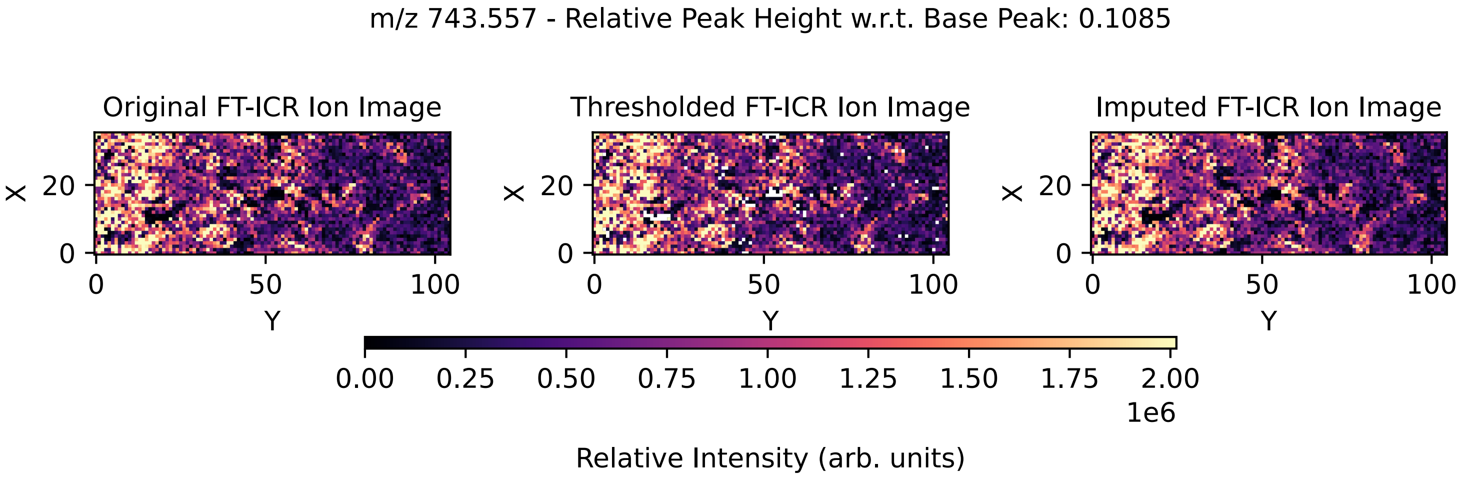

Figure 7: Raw, thresholded and imputed ion image of m/z-bin 743.557. This is a relatively high-intensity peak w.r.t. the base peak (0.1085 of the base peak height). The right image shows visually good imputation when compared to the original ion image.

4.1 Post-Processing Example

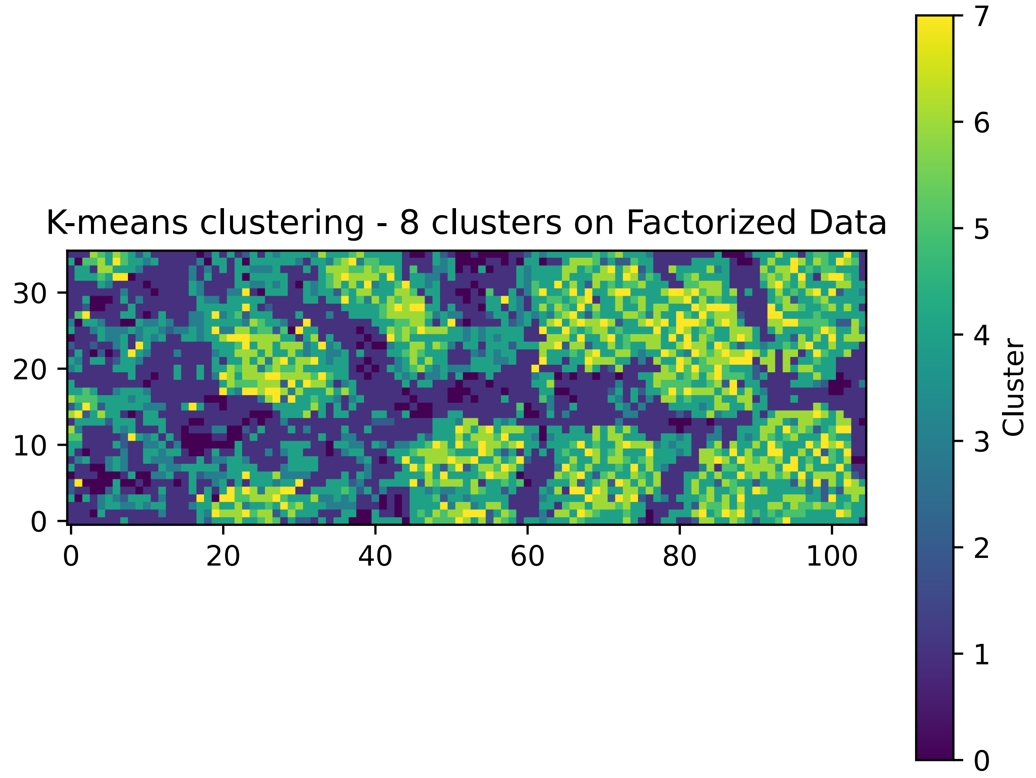

Figure 8: k-means clustering with 8 clusters applied to factorized FT-ICR data. The visualization of clusters, segmenting the tissue in the process, shows good correspondence to (presumed to be biological) patterns also visible in the total ion image.

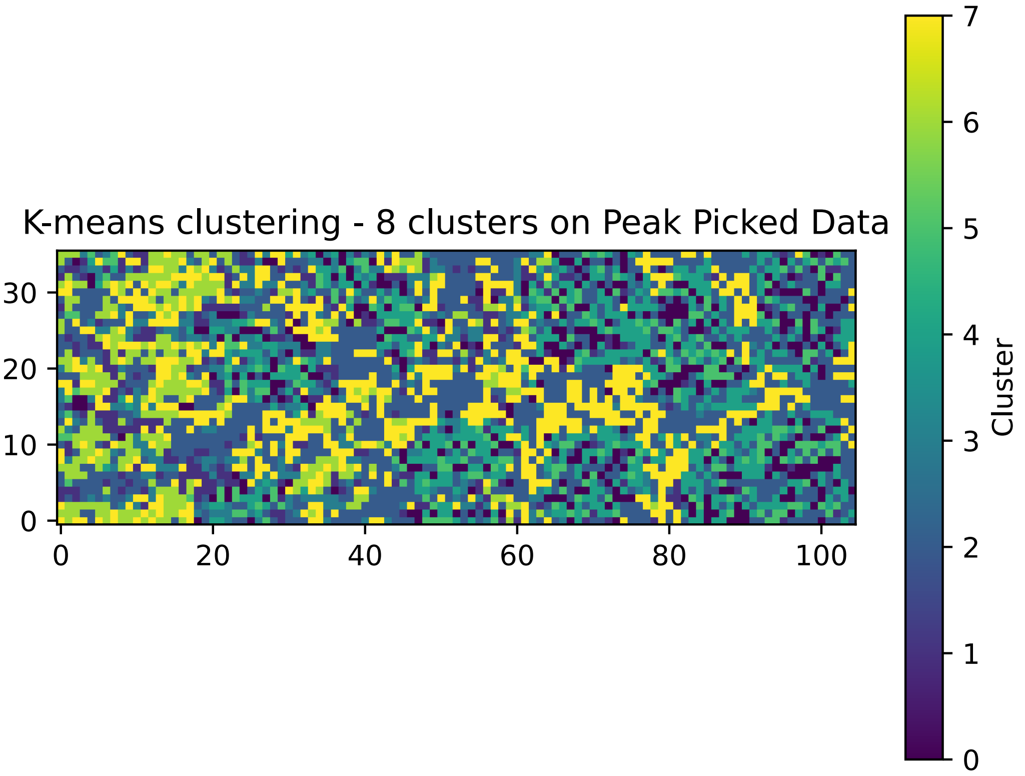

Figure 9: k-means clustering with 8 clusters applied to raw peak picked (100 peaks) FT-ICR data. The visualization of clusters, segmenting the tissue, shows some correspondence with respect to the total ion image, but less compared to the results on factorized FT-ICR data (Fig. 8).

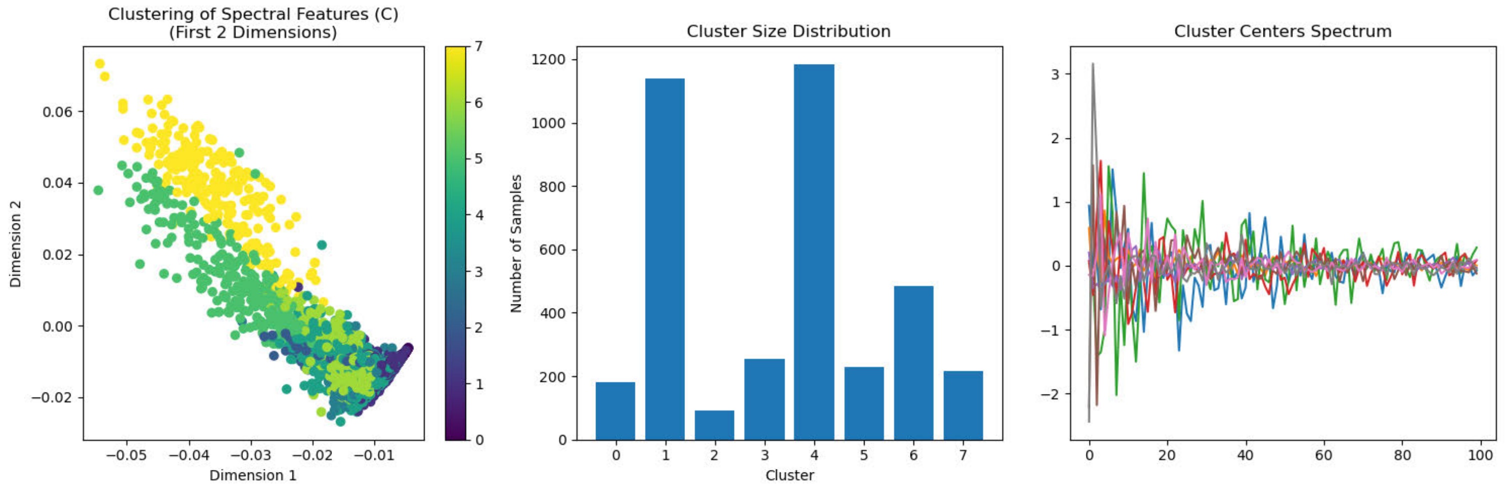

Figure 10: k-means clustering with 8 clusters applied to the factorized FT-ICR data. We provide some further details on the results shown in Fig. 8, namely a visualization of the cluster-labeled data points along the first two latent dimensions, the distribution of cluster sizes, and the centers of cluster spectra.

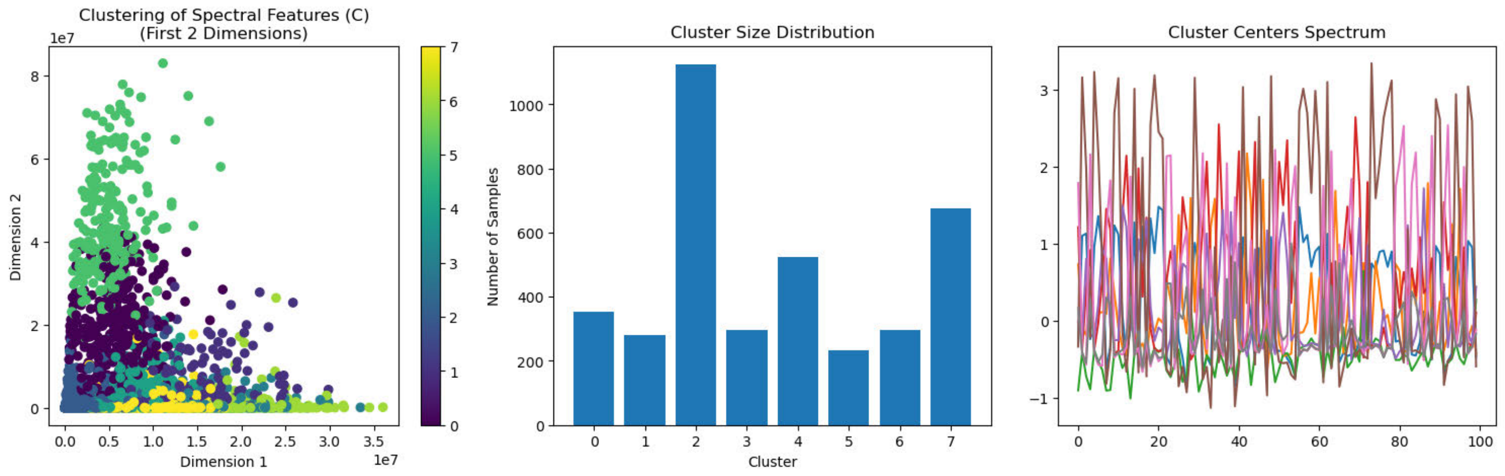

Figure 11: k-means clustering with 8 clusters applied to the raw peak picked (100 peaks) FT-ICR data. We provide some further details on the results shown in Fig. 9, namely a visualization of the cluster-labeled data points along the first two latent dimensions, the distribution of cluster sizes, and the centers of cluster spectra.

5 Case Study 3: Advantages and Disadvantages of Low-Rank Matrix Completion for Missing Value TOF IMS Data

5.1 Ion Image Examples

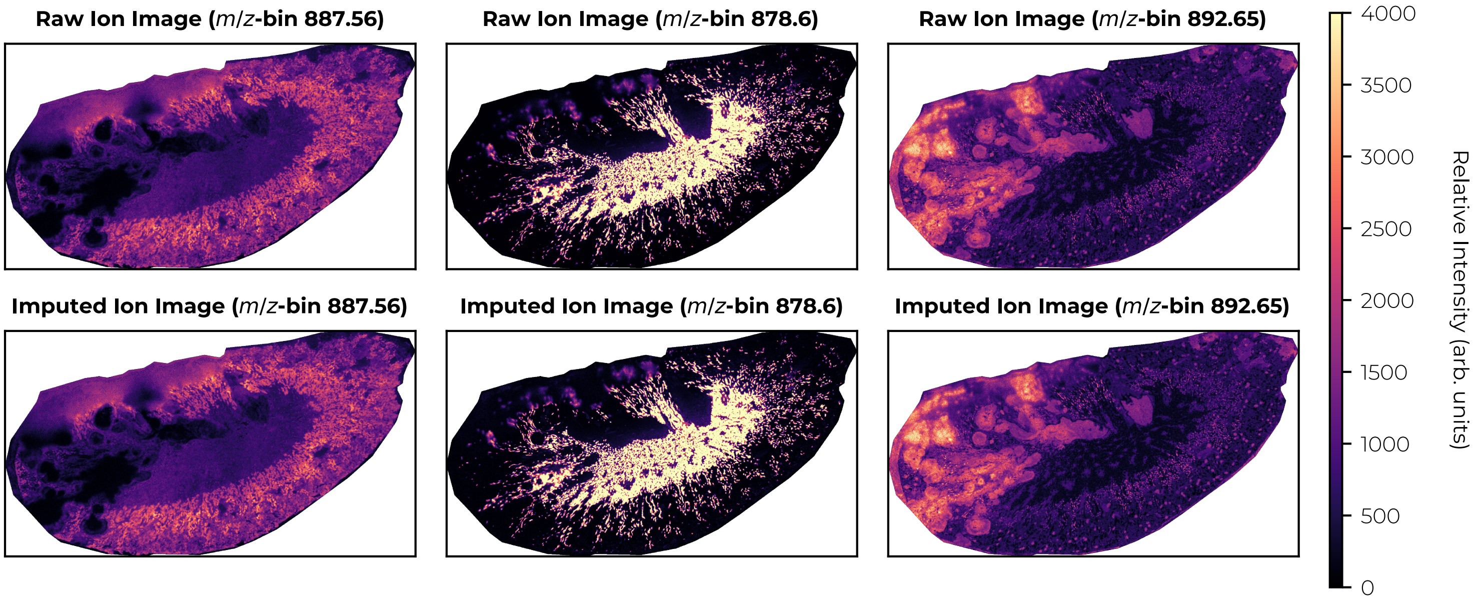

Figure 12: In the first row, the three columns depict different ion images retrieved from the raw data, namely m/z 887.56, m/z 878.60 and m/z 892.65. These ion species are approximated by the SVT, and shown in the second row. These ion images are shown as representatives of high-intensity peaks.

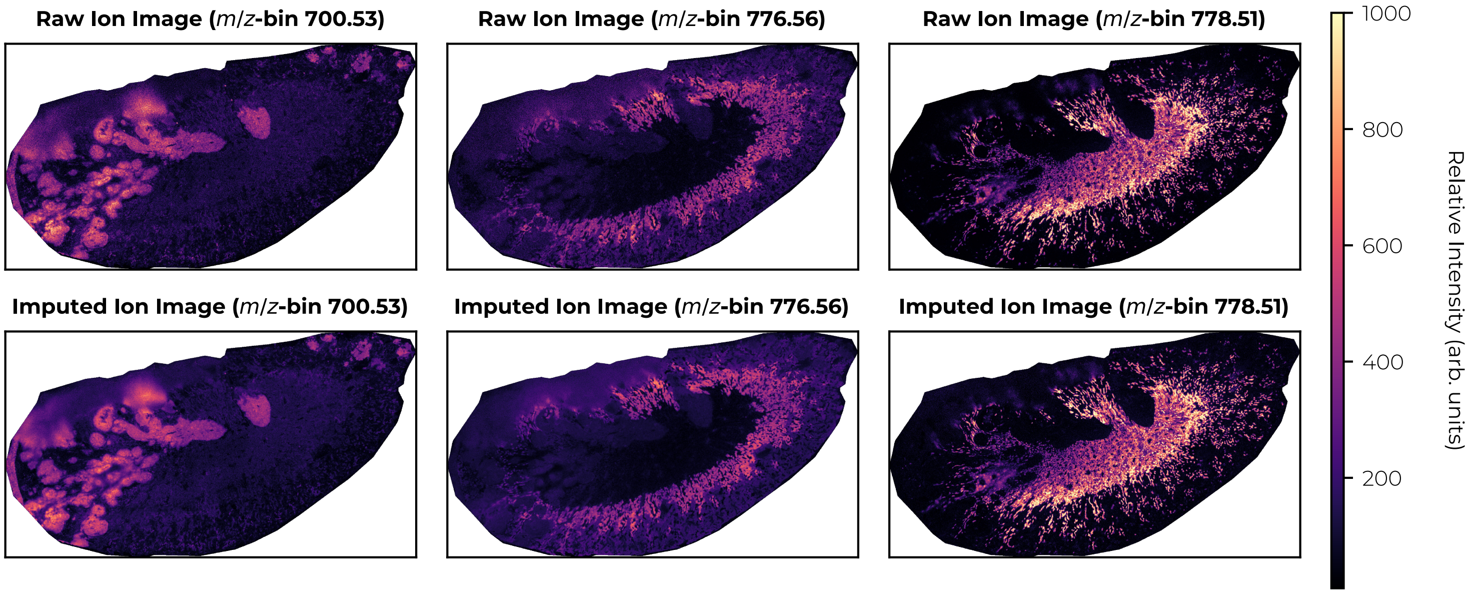

Figure 13: In the first row, the three columns depict different ion images retrieved from the raw data, namely m/z 700.53, m/z 776.56 and m/z 778.51. These ion species are approximated by the SVT, and shown in the second row. These ion images are shown as representatives of lower-intensity peaks.

5.2 Spectral Error Distribution and Biological Interpretation

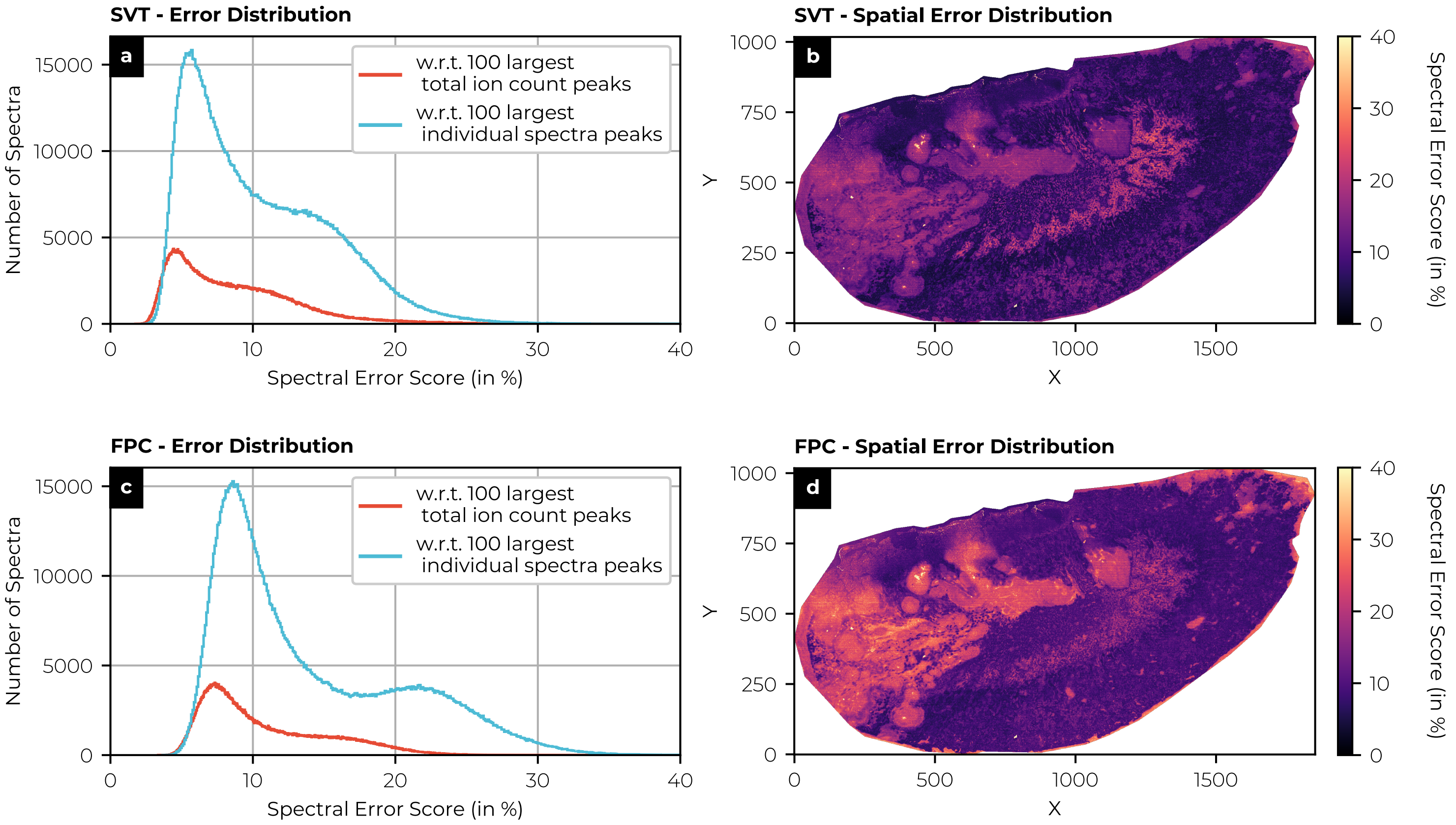

Figure 14: Spectral error score, *i.e.*, $\frac{||\tilde{M}_{i\bullet}-X_{i\bullet}||_2}{||\tilde{M}_{i\bullet}||_2}$, reports on the error of individual spectra. The distribution of the spectral error score is given (left) for both SVT and FPC methods with respect to the 100 largest total ion current count peaks and with respect to the 100 largest individual spectral peaks. The spatial distribution for the spectral error score with respect to the 100 largest individual spectra peaks is also depicted for the SVT and FPC (right).

5.3 Methodological Effects on Reconstructed Ion Images and Spectra

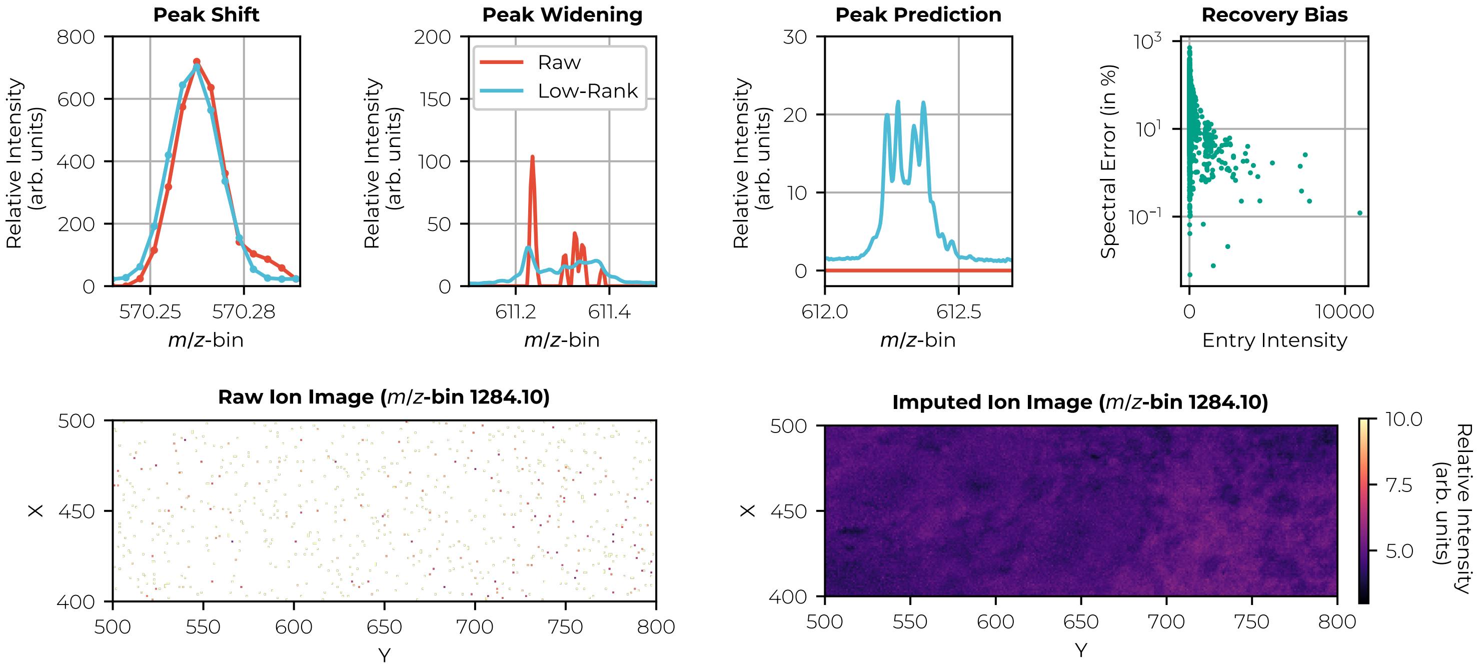

Figure 15: The first row depicts four potential effects of the low-rank approximation of a complete dataset on individual spectra, namely peak shifting (*i.e.*, small shifts of the peak center), peak widening, peak prediction (potential introduction of low-intensity peaks in line with patterns observed in the rest of the dataset), and bias in the recovery error for large peaks (better recovery/representation of large peaks). In the second row, an extreme example is given of an individual ion image at m/z 1284.10, both raw (left) and recovered (right). The predicted image depicts a biological scene, even though the raw image contains barely any data points (very sparse). The missing values in the image are imputed, based on the available information from the rest of the dataset. This could lead to a substantial imputation error for those ion species. These effects are a price we pay for retaining full spectrum information in our dimensionality reduction methods, and they affect low-abundant and missing pixel-dominated species first. As such, we robustly keep the full spectrum profile intact for higher intensity peaks, in contrast to selective peak picking. At the same time, these recognized shortcomings can be taken into account for future model improvements.

5.4 Preservation of Near-Isobaric Species

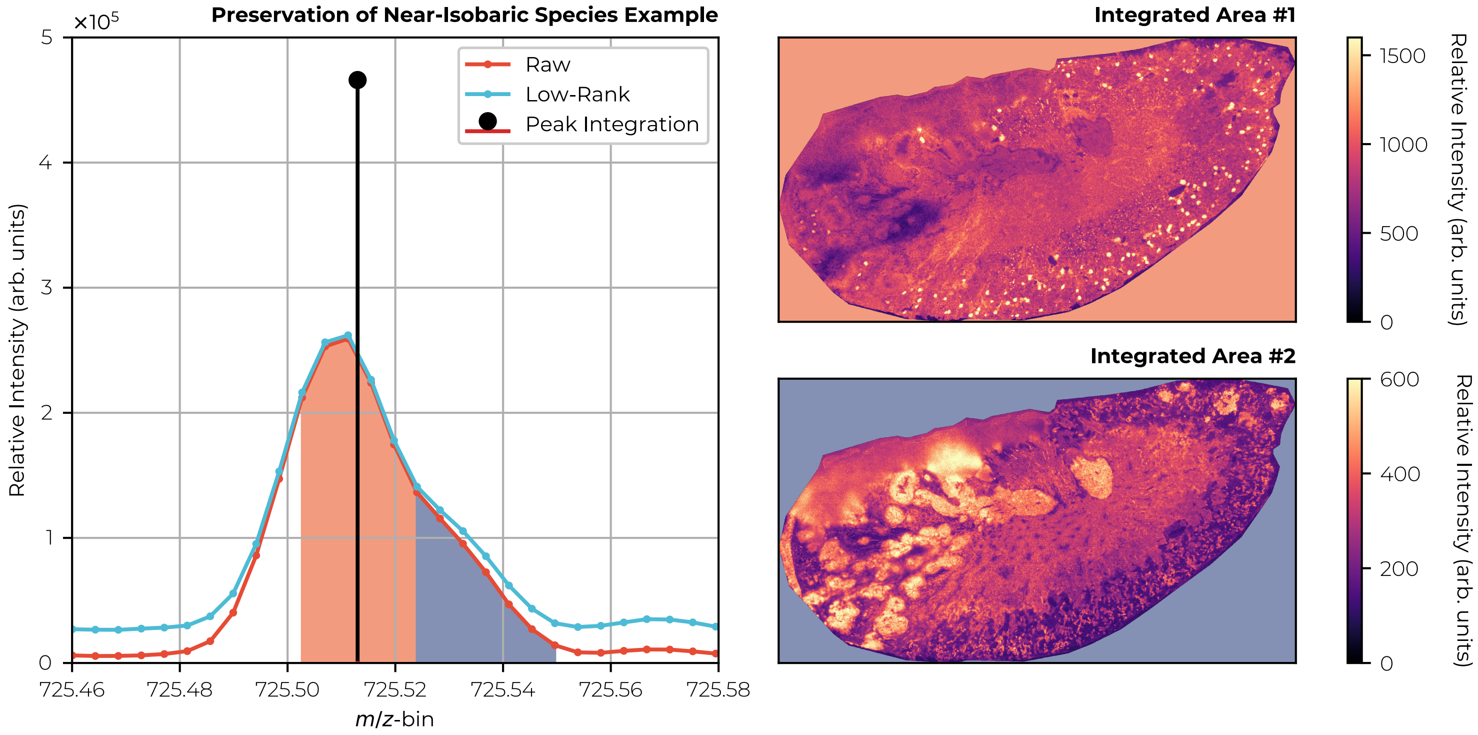

Figure 16: A particular spectrum is examined (left) in the range between m/z 725.46 and 725.58 from the perspective of both the low-rank approximation and raw data, along with a peak integrated version. The highlighted (orange and blue) areas under the curve are integrated and spatially depicted (right). We observe that the shoulder (blue) differs spatially from the peak (orange). With peak integration, relative information of the shoulder (blue) is lost, due to its weak signal.

5.5 Retention of Lower-Intensity Ion Species and Bias Mitigation

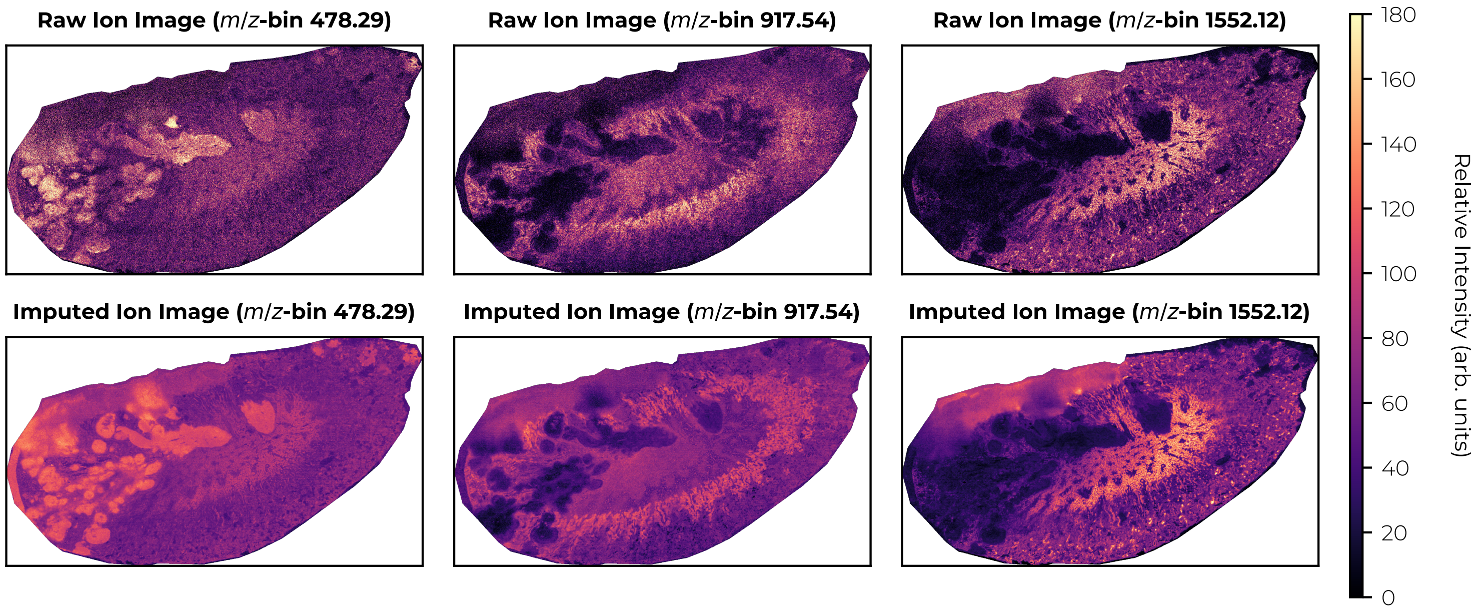

Figure 17: In the first row, the three columns depict different ion images as found in the raw data, namely m/z 479.29, m/z 917.54 and m/z 1552.12. These ion species are detected in more than 80% of the IMS pixels, they are not isotopes, and their distributions suggest a biology-driven distribution. Nevertheless, they would be disregarded in a simple peak picking procedure, if *e.g.,* only the 1000 largest peaks would be retained. Our approach (see second row) retains these ion species, albeit approximately.

6 Sample Preparation: Human Kidney Tissue (FT-ICR IMS)

Human kidney tissue was surgically removed during a full nephrectomy, and remnant tissue was processed for research purposes by the Cooperative Human Tissue Network (CHTN) at Vanderbilt University Medical Center. Human biospecimens were collected in compliance with the CHTN protocols, institutional IRB policies, and the National Cancer Institute's best practices for procurement of remnant surgical research material. The tissue was flash-frozen over an isopentane-dry ice slurry and embedded in carboxymethylcellulose. Tissue sections were cryosectioned with a thickness of 10 μm and thaw-mounted onto indium tin-oxide-coated glass slides (Delta Technologies, Loveland, CO, USA). 1,5-Diaminonaphthalene (DAN) was applied to the tissue surface using a TM Sprayer M3 (HTX Technologies, Chapel Hill, NC, USA). The sample was imaged (50 μm pitch) directly after matrix

Table 1: SVT and FPC, with random sampling scheme β. A moderate reconstruction error is observed for all methods for both raw and low-rank input and references. The imputation error is substantial. For sampling scheme β, the impact of the imputation error is larger on the global error. For SVT, we set parameters $\delta = 1$ and $\tau = 10^{-3}$ and for FPC, we set $\delta = 1.4$ and $\tau = 10^{-3}$ (see Supplementary Materials 9). For SVT with raw input data we obtain a 111 rank solution and for FPC a 111 rank solution. We truncate all solutions to a rank of 100 for fair comparison. The SVT took on average 106 minutes to converge, the FPC algorithm on average 19 minutes.

**Input** M	**Reference** $\tilde{M}$	**Method**	**Rank**	**Reconstruction Error** $\frac{\|\|P_\Omega(\tilde{M}-X)\|\|_F}{\|\|P_\Omega(\tilde{M})\|\|_F} \times 100\%$	**Imputation Error** $\frac{\|\|P_{\Omega_c}(\tilde{M}-X)\|\|_F}{\|\|P_{\Omega_c}(\tilde{M})\|\|_F} \times 100\%$	**Global Error** $\frac{\|\|\tilde{M}-X\|\|_F}{\|\|\tilde{M}\|\|_F} \times 100\%$
Raw	Raw	SVT	100	50.0	92.6	89.6
Raw	Raw	FPC	100	29.3	84.9	81.6
Raw	Low-Rank	SVT	100	48.3	92.2	89.2
Raw	Low-Rank	FPC	100	23.5	84.2	80.7
Low-Rank	Low-Rank	SVT	100	67.9	92.4	90.5
Low-Rank	Low-Rank	FPC	100	22.8	84.4	80.9

application with a 15T MALDI Fourier-transform ion cyclotron resonance (FT-ICR) mass spectrometer (Solarix, Bruker Daltonics, Billerica, MD, USA). Briefly, data were generated from m/z 300-2000 with a 4M file size in negative ion mode.

7 Sample Preparation: Mouse Kidney Tissue (qTOF IMS)

C57BL6/J mice were retro-orbitally infected with *S. aureus* Newman and sacrificed humanely five days post-infection. Kidneys were flash frozen on an isopentane/dry ice slurry and embedded in 2.6% carboxymethylcellulose. 5 μm thick sections were collected using a Leica Biosystems CM3050S cryostat and thaw-mounted onto indium tin oxide coated glass slides. Sections were washed with cold 150 mM ammonium formate for 45 seconds for three total washes. 5 mg of 4-(dimethylamino)cinnamic acid matrix was applied using an in-house sublimation device. MALDI IMS data were collected in negative ion mode from m/z 400-2000 using a Bruker MALDI timsTOF fleX platform with a 5 μm step size, 25% laser power, and 25 shots per pixel.

8 Data Preprocessing

The TOF dataset was m/z-aligned with a custom program [Migas, 2024, Monchamp et al., 2007, Farrow et al., 2022]. For all case studies, we used a 5-95% TIC pixel normalization method, *i.e.,* scaling each row by the sum of its entries between 5%-percentile and 95%-percentile [Migas, 2024, Monchamp et al., 2007, Farrow et al., 2022]. We did not statistically normalize (*i.e.*, mean subtraction and scaling) the features, *i.e.,* m/z-bins (columns), as we observed that it hindered low-rank recovery, presumably due to the majority of measured m/z-bins consisting of low signal-to-noise measurements, sub-noise features or noise features. Statistically normalizing the m/z-bins amplifies the singular values associated with the noise subspace while diminishing those linked to the signal subspace, particularly in high-intensity m/z-bins. Further exploration of advanced normalization schemes was deemed beyond the scope of this paper. All calculations were performed on a Dell Precision 7920 workstation with 56 cores at 2.7 GHz and 1.5 TB of memory, and two NVIDIA A6000 GPUs connected via NVLink Bridge.

9 Parameter Setting

For SVT, convergence for the completion problem is guaranteed if $0 < \delta < 2$ [Cai et al., 2010]. However, it was also noted that this choice can be too conservative, and the convergence slow [Cai et al., 2010]. For our datasets, we found that setting the parameter to $\delta > 2$ breaks the convergence and that values of $\delta < 1$ lead to slow convergence. Hence, we manually tuned the values per experiment between $1 < \delta < 1.7$. For FPC, different suggestions are made for setting δ [Candes and Plan, 2010, Ma et al., 2011]. We found by manually tuning that convergence was ensured for our datasets settings values, similar to SVT, between $1 < \delta < 2$.

Although different suggestions for SVT's and FPC's τ exist (note that τ is described by μ for FPC [Candes and Plan, 2010]), we defined τ relative to $\max(m, n)$. By trial-and-error, we found values between 10^{-1} and 10^{-3} perform adequate to obtain low-rank solutions. We did not observe large deviations in recovery when setting the parameters in those ranges. A parameter sensitivity analysis is, however, advisable to further tune the outcomes.

For the divide, factor and conquer approach, the raw data matrix $M \in \mathbb{R}^{312,249 \times 1,372,421}$ was divided into $3,303$ subsampled matrices, $C_i \in \mathbb{R}^{312,249 \times 400}$, where $i \in [1, 3303]$. Subsampling was performed along the spatial axis, which outperformed spectral axis subsampling due to the presence of many noise-dominant m/z-bins. Spectral subsampling risked aggregating noise features into the same subsampled matrices, leading to faulty results. Sampling sizes of $300 - 500$ spectra were heuristically found to ensure good recovery, *i.e.,* fulfilling the low-rank constraint while allowing for efficient computation, completing within a 12-hour runtime.

Table 2: Robustness analysis of SVT and FPC, with random sampling scheme β through random subsampling (subsampling here 20% of the rows, *i.e.* pixels, rather than 8.9% to ensure a speed-up in calculation for the different iterations). We observe the same phenomena as in Table 1 with very little variation. The results are provided as $\mu \pm \sigma$, where μ is the mean and σ is the standard deviation over 3 runs). The small variation suggests that the sampling and subsampling are quite representative for the dataset distribution. The change in mean compared to Table 1 is mostly caused by parameter settings with respect to, *e.g.*, dataset size. For SVT, we set parameters $\delta = 1$ and $\tau = 10^{-3}$ and for FPC, we set $\delta = 1.4$ and $\tau = 10^{-3}$ (see Supplementary Materials 9). We stopped the convergence early at a rank of 100. For SVT with raw input data we obtain a 111 rank solution and for FPC a 111 rank solution. For SVT with low-rank input data, we obtain a 111-rank solution and, for FPC, a 111-rank solution. We truncate all solutions to a rank of 100 for fair comparison.

**Input** M	**Reference** $\tilde{M}$	**Method**	**Rank**	**Reconstruction Error** $\frac{\|\|P_\Omega(\tilde{M}-X)\|\|_F}{\|\|P_\Omega(\tilde{M})\|\|_F} \times 100\%$	**Imputation Error** $\frac{\|\|P_{\Omega_c}(\tilde{M}-X)\|\|_F}{\|\|P_{\Omega_c}(\tilde{M})\|\|_F} \times 100\%$	**Global Error** $\frac{\|\|\tilde{M}-X\|\|_F}{\|\|\tilde{M}\|\|_F} \times 100\%$
Raw	Raw	SVT	100	40.08 ± 0.024	97.34 ± 0.0002	94.21 ± 0.0007
Raw	Raw	FPC	100	21.08 ± 0.0008	90.98 ± 0.0001	87.61 ± 0.0003
Raw	Low-Rank	SVT	100	41.08 ± 0.024	99.06 ± 0.0002	95.80 ± 0.0006
Raw	Low-Rank	FPC	100	19.96 ± 0.0006	92.39 ± 0.0002	88.85 ± 0.0006
Low-Rank	Low-Rank	SVT	100	36.01 ± 0.03	98.82 ± 0.0002	95.42 ± 0.0006
Low-Rank	Low-Rank	FPC	100	15.83 ± 0.0004	92.14 ± 0.0002	88.56 ± 0.0007

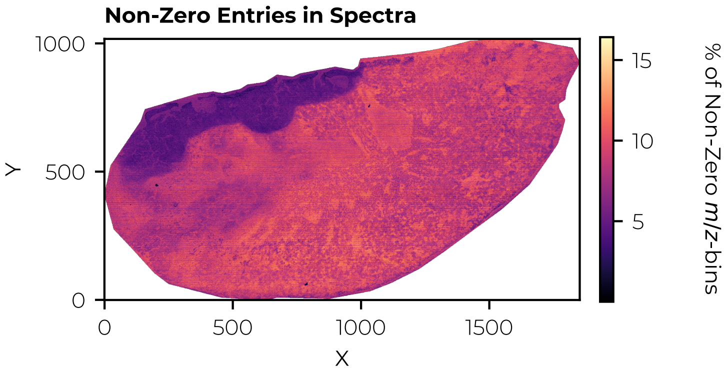

Figure 18: Spatial distribution of the number of non-zero values per spectrum. Dark regions correspond to spectra with few values (after clipping), while bright regions reflect spectra with more measured values. Note that most spectra only contain $2 - 30\%$ of measured values. Some spatial regions contain more zeroes, *i.e.,* fewer features are captured.

References

T. Alexandrov. Maldi imaging mass spectrometry: statistical data analysis and current computational challenges. *BMC bioinformatics*, 13(Suppl 16):S11, 2012.

D. M. Anderson, R. Van de Plas, K. L. Rose, S. Hill, K. L. Schey, A. C. Solga, D. H. Gutmann, and R. M. Caprioli. 3-d imaging mass spectrometry of protein distributions in mouse neurofibromatosis 1 (nf1)-associated optic glioma. *Journal of proteomics*, 149:77–84, 2016.

J.-F. Cai, E. J. Candès, and Z. Shen. A singular value thresholding algorithm for matrix completion. *SIAM Journal on optimization*, 20(4):1956–1982, 2010.

E. J. Candes and Y. Plan. Matrix completion with noise. *Proceedings of the IEEE*, 98(6):925–936, 2010.

J. Dongarra, M. Gates, A. Haidar, J. Kurzak, P. Luszczek, S. Tomov, and I. Yamazaki. The singular value decomposition: Anatomy of optimizing an algorithm for extreme scale. *SIAM review*, 60(4):808–865, 2018.

M. A. Farrow, L. E. Tideman, E. K. Neumann, N. H. Patterson, L. G. Migas, M. E. Colley, J. L. Allen, E. S. Rivera, C. E. Romer, H. Yang, et al. A lipid atlas of the human kidney. *bioRxiv*, pages 2022–04, 2022.

A. González-Fernández, A. Dexter, C. J. Nikula, and J. Bunch. Nectar: A new algorithm for characterizing and correcting noise in qtof-mass spectrometry imaging data. *Journal of the American Society for Mass Spectrometry*, 34(11):2443–2453, 2023.

N. Halko, P.-G. Martinsson, and J. A. Tropp. Finding structure with randomness: Probabilistic algorithms for constructing approximate matrix decompositions. *SIAM review*, 53(2):217–288, 2011.

S. Ma, D. Goldfarb, and L. Chen. Fixed point and bregman iterative methods for matrix rank minimization. *Mathematical Programming*, 128(1):321–353, 2011.

L. W. Mackey, A. Talwalkar, and M. I. Jordan. Distributed matrix completion and robust factorization. *J. Mach. Learn. Res.*, 16 (1):913–960, 2015.

L. G. Migas. msalign: Spectral alignment based on matlab's 'msalign' function. *0.2.0*, 2024. URL `https://github.com/lukasz-migas/msalign`.

P. Monchamp, L. Andrade-Cetto, J. Y. Zhang, and R. Henson. Signal processing methods for mass spectrometry. *Systems Bioinformatics: An Engineering Case-Based Approach, Artech House Publishers*, 2007.

N. Verbeeck, R. M. Caprioli, and R. Van de Plas. Unsupervised machine learning for exploratory data analysis in imaging mass spectrometry. *Mass spectrometry reviews*, 39(3):245–291, 2020.

T. Zhou and D. Tao. Godec: Randomized low-rank & sparse matrix decomposition in noisy case. In *Proceedings of the 28th International Conference on Machine Learning, ICML 2011*, 2011.
